# Optimization of Potent and Selective Cyclohexyl Acid ERAP1 Inhibitors Using Structure- and Property-Based Drug Design

**DOI:** 10.1021/acsmedchemlett.4c00401

**Published:** 2024-11-06

**Authors:** Ross P. Hryczanek, Andrew S. Hackett, Paul Rowland, Chun-wa Chung, Máire A. Convery, Duncan S. Holmes, Jonathan P. Hutchinson, Semra Kitchen, Justyna Korczynska, Robert P. Law, Jonathan D. Lea, John Liddle, Richard Lonsdale, Margarete Neu, Leng Nickels, Alex Phillipou, James E. Rowedder, Jessica L. Schneck, Paul Scott-Stevens, Hester Sheehan, Chloe L. Tayler, Ioannis Temponeras, Christopher P. Tinworth, Ann L. Walker, Justyna Wojno-Picon, Robert J. Young, David M. Lindsay, Efstratios Stratikos

**Affiliations:** # GSK, Medicines Research Centre, Gunnels Wood Road, Stevenage SG1 2NY, U.K.; † National Center for Scientific Research “Demokritos”, Agia Paraskevi, Attiki 15341, Greece; ‡ Department of Pure and Applied Chemistry, University of Strathclyde, Glasgow G1 1XL, U.K.

**Keywords:** ERAP1, immunotherapy, structure-based drug design, property-based drug design, ligand efficiency, lipophilic ligand efficiency, FEP

## Abstract

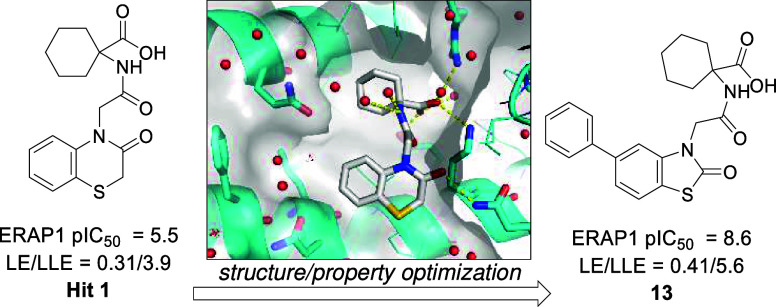

Endoplasmic reticulum aminopeptidase 1 (ERAP1) cleaves the *N*-terminal amino acids of peptides, which can then bind onto major histocompatibility class I (MHC-I) molecules for presentation onto the cell surface, driving the activation of adaptive immune responses. In cancer, overtrimming of mature antigenic peptides can reduce cytotoxic T-cell responses, and ERAP1 can generate self-antigenic peptides which contribute to autoimmune cellular responses. Therefore, modulation of ERAP1 activity has potential therapeutic indications for cancer immunotherapy and in autoimmune disease. Herein we describe the hit-to-lead optimization of a series of cyclohexyl acid ERAP1 inhibitors, found by X-ray crystallography to bind at an allosteric regulatory site. Structure-based drug design enabled a >1,000-fold increase in ERAP1 enzymatic and cellular activity, resulting in potent and selective tool molecules. For lead compound **7**, rat pharmacokinetic properties showed moderate unbound clearance and oral bioavailability, thus highlighting the promise of the series for further optimization.

Endoplasmic reticulum aminopeptidase 1 (ERAP1) cleaves *N*-terminal amino acids from antigenic peptide precursors for the downstream interaction with the adaptive immune system. Amino acids are sequentially cleaved from peptide lengths of up to 16 amino acid residues down to 8–10-mer peptides.^1^ After trimming, these peptides are loaded onto MHC-I molecules, which are transported to the surface of the cell.^1−3^ The complex composition of the antigenic peptides presented constitutes a representation of the state of the cell to the extracellular environment, which includes any potential malfunctions.^3^ The peptide–MHC-I complexes are recognized by cells of the adaptive immune system response, such as cytotoxic T-cells, and this can induce apoptosis for infected or aberrant cells.^3^

Given that ERAP1-trimmed peptides modulate the adaptive immune response, controlling ERAP1 activity is an attractive strategy for cancer immunotherapy, autoimmune diseases and increasing immune responses against pathogens.^4^ There is evidence of upregulation of ERAP1 in cancerous cells,^4^ where peptides can be “overtrimmed” and thus cannot be presented in a complex with MHC-I, which has strict length requirements.^5^ This effectively hides the cancerous cells from the immune system. Therefore, it is thought that inhibition of ERAP1 could prevent this “overtrimming” and thus highlight cancerous cells to the immune system that would have otherwise been hidden by up-regulated ERAP1.

A focus of developing ERAP1 modulators has been achieving selectivity over the closely related enzymes endoplasmic reticulum amino peptidase 2 (ERAP2) and insulin-regulated aminopeptidase (IRAP).^6−9^ ERAP1, ERAP2 and IRAP have highly conserved active sites and around 50% sequence identities.^6^ ERAP2 has a substrate preference different from that of ERAP1 but a similar function in regulating the adaptive immune response. IRAP also has a similar function to ERAP1 and, additionally, regulates both glucose uptake into cells and oxytocin levels in pregnancy.^10^

Currently, there are no approved ERAP1 inhibitor therapeutics; therefore, there is a need to develop highly potent and selective inhibitors with good pharmacokinetic exposure.^11^ Potent phosphinic pseudotripeptide ERAP1 inhibitors with limited ERAP2 and IRAP selectivity have been reported,^3,7^ as have selective molecules with lower ERAP1 inhibition potency.^6,8,9^ A series of benzofuran carboxylic acids with nanomolar potency were found to be selective over ERAP2 but were not tested against IRAP (Figure 1).^12^ A series of phenyl-sulfamoyl benzoic acid inhibitors from Gray Wolf Therapeutics have been reported with nanomolar activity.^13^ A compound from this series, GRWD5769, is currently in phase 1/2 clinical trials for patients with advanced solid tumors.^11^

**Figure 1:**
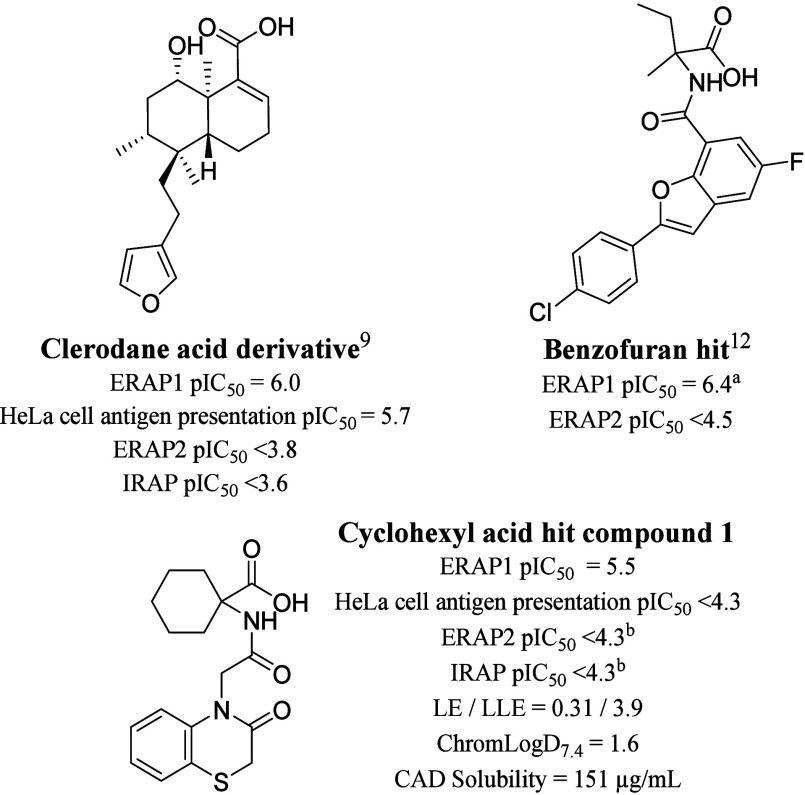
Profiling data for previously described clerodane acid derivative^9^ and benzofuran hit.^12^ This work describes the development of the cyclohexyl acid hit compound **1**. Peptidic substrates: ERAP1 (YTAFTIPSI or ^*a*^L-Rho-Succ-FKARKF), HeLa cell antigen presentation (LEQLESIINFEKL), ERAP2 (Arg-AMC), IRAP (Leu-AMC). ^*b*^*n* = 1.

Within our laboratories, the natural product clerodane acid derivative (Figure 1) was identified from a high-throughput screen for ERAP1 modulators.^9^ This compound activates the hydrolysis of the small fluorogenic substrate Leu-AMC 2–2.5-fold yet inhibits the cleavage of antigenic epitope YTAFTIPSI (pIC_50_ = 6.0). The clerodane acid derivative also showed excellent selectivity over ERAP2 and IRAP and was active in regulating the immunopeptidome of a melanoma cancer cell line.^14^ We were encouraged by the clerodane acid derivative results but sought to develop alternative chemical matter with increased synthetic tractability. The objective was to find ERAP1 inhibitors, using physiologically relevant antigenic peptide substrates, working toward the “overtrimming” hypothesis, where it is thought that ERAP1 may destroy mature antigenic peptides of cancer cells.^4^

Also discovered in the same high-throughput screen as the clerodane acid derivative was the hit cyclohexyl acid compound **1**, which had ERAP1 inhibitory potency and a selectivity profile similar to those of the clerodane acid derivative (Figure 1). Hit compound **1** was found by X-ray crystallography to bind at the same regulatory site as the clerodane acid derivative (Figure 2). Compound **1** had reduced structural complexity compared to the clerodane acid derivative and was therefore considered a more synthetically tractable molecule for hit-to-lead optimization. The structure, efficiency, and physical properties of compound **1** represented an attractive starting point; the structure- and property-based optimization of compound **1** is the focus of this work.

**Figure 2:**
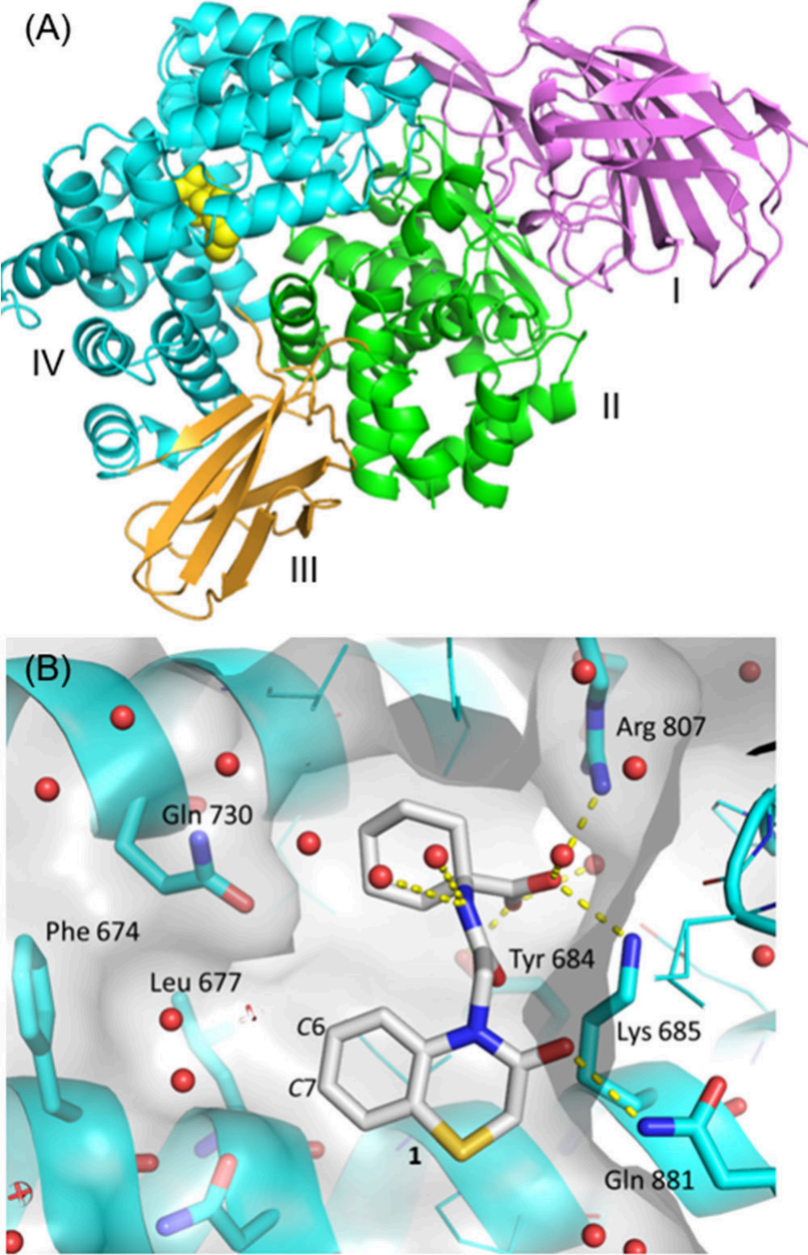
X-ray cocrystal structure of cyclohexyl acid compound **1** bound to ERAP1. Resolution 1.72 Å, PDB: 9GJN. (A) Compound **1** (yellow sphere), domain I (pink), domain II (green), domain III (gold), domain IV (cyan). (B) Compound **1** in binding site (gray)

Enzymatic ERAP1 activity was assessed using a high-throughput mass spectrometry assay that measures the cleavage of the 9-amino acid peptide YTAFTIPSI to form TAFTIPSI, as described previously.^9^ This antigenic natural epitope precursor was derived from the human immunodeficiency virus^15^ and was chosen as it is a highly ERAP1-sensitive substrate.^6^ In this assay, compound **1** had an ERAP1 pIC_50_ value of 5.5. Cellular activity was measured using a flow cytometry-based assay that measures the presentation of the antigenic epitope SIINFEKL,^9^ and compound **1** did not show measurable inhibitory activity in this assay. Compound **1** has a ChromLogD_7.4_^16^ value of 1.6, and this low lipophilicity could result in poor cell membrane permeability, which could explain the lack of cellular activity.^17^ Thus, in addition to increasing potency, a primary objective of this work was to increase the lipophilicity of hit compound **1** with the aim of achieving cellular potency. The charge and low lipophilicity of compound **1** resulted in high aqueous solubility based on a kinetic CAD measurement.^18,19^

We recognized the opportunity to increase lipophilicity but aimed to do this in a controlled and efficient manner, and therefore we proactively pursued increases in lipophilic ligand efficiency (LLE; Figure 1).^20^ We used a variation of LLE defined as ERAP1 pIC_50_ – ChromLogD_7.4_.^16^ In addition, ligand efficiency (LE) was also tracked to ensure heavy-atom count was being used efficiently.^20^ The benefits of optimizing LE and LLE have been discussed extensively^21−25^ and include improved target selectivity, improved ADMET properties and greater design flexibility throughout optimization. Further, marketed drug molecules tend to have superior LE and LLE values compared to published nondrug molecules.^20,25^ For this work, specific target values for LE and LLE were not chosen; instead, the objective was to simply increase LE and LLE from their values in hit **1**. Ultimately, increasing potency in a lipophilicity and heavy-atom efficient manner should yield higher quality leads with greater lipophilicity and heavy-atom flexibility for any potential future lead optimization.

Three major binding interactions were observed from the carboxylic acid of compound **1** to Arg807, Lys685 and Tyr684 (Figure 2). These key interactions are thought to be where the *C*-termini of endogenous peptides bind.^26^ Binding of peptides to the regulatory site plays a critical role in determining ERAP1 substrate length requirements and the extent to which peptide trimming occurs through a “molecular ruler” mechanism.^2^ The cyclohexane ring occupied a lipophilic pocket, and the attached amide did not clearly show any specific polar interactions with the protein. An additional hydrogen bond interaction was observed from the carbonyl of the thiomorpholinone motif with Gln881. Adjacent to the aromatic ring of the benzothiomorpholinone was a pocket containing lipophilic residues Phe674 and Leu677, shown to be occupied by a water molecule. Previously reported benzofuran compounds that contained examples featuring the same cyclohexyl acid-amide motif were predicted by docking studies to have similar binding, though they did not make the hydrogen-bonding interaction with Gln881.^12^

Based on the binding of compound **1**, molecules with systematic single-atom point changes were designed and synthesized (Table 1), with the aim of increasing ERAP1 potency and potentially achieving cellular activity by the introduction of lipophilic motifs. The first area investigated was the substitution of the bicyclic motif, as the X-ray cocrystal structure indicated potential space for growth into the lipophilic pocket containing Phe674 and Leu677 (Figure 2). The *C*6 and *C*7 positions of the benzothiomorpholinone ring did not appear as ideal vectors to grow into this space; however, it was hypothesized that if movement in the protein and ligand was tolerated, then this substitution may increase ERAP1 potency.

**Table 1:**
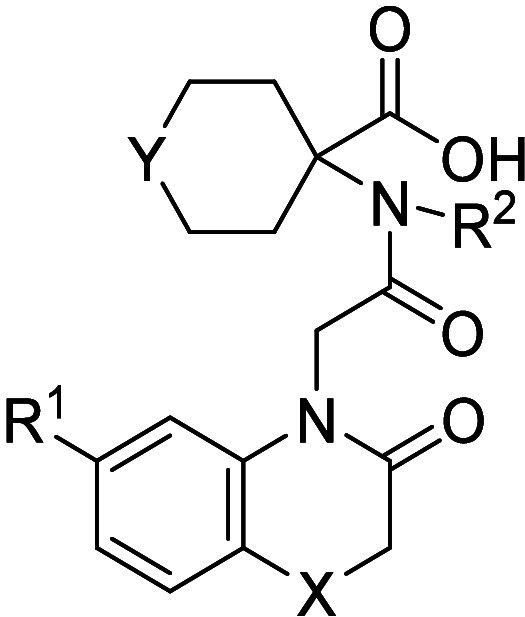

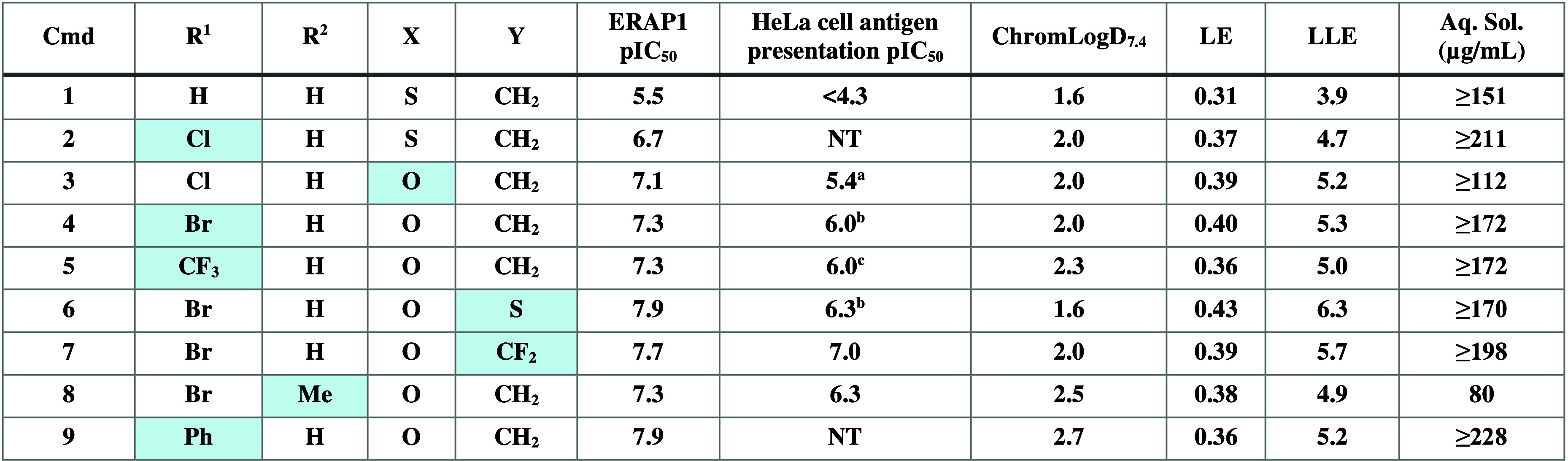
Early SAR Investigations of Systematic Point Changes to Hit 1 ERAP1 and Cell Potency (Data Are *n* = 3 or Higher unless Otherwise Stated)

a Tested at <4.3 on two test occasions.

b *n* = 2.

c Tested at <4.3 on one test occasion. NT = not tested

Halogenation of the *C*6 position resulted in chloro-substituted compound **2**, which was over 10-fold more potent compared to the hit compound **1** in the enzymatic assay. Subsequently, the thiomorpholinone motif was investigated, with exchange of the sulfur atom for an oxygen also leading to increased potency, as shown by morpholinone **3**. Alternative lipophilic substitutions at the *C*6 position of the benzothiomorpholinone motif, such as bromide derivative **4** and trifluoromethyl analogue **5**, were well tolerated. Modified cyclohexane rings, such as sulfide **6** and difluorocyclohexane **7**, resulted in further increases in potency. Methylation of the amide, giving compound **8**, was potency neutral compared to the N–H matched pair **4**, which was somewhat expected, given that the N–H did not appear to make any specific interactions with the protein and pointed toward solvent in the X-ray crystal structure. This change did, however, increase lipophilicity and remove a hydrogen bond donor, and therefore it was considered a useful physicochemical property modulation handle. Finally, phenyl-substituted compound **9** demonstrated that large lipophilic groups were well tolerated, suggesting significant movement in the binding site or ligand. Without significant movement of the protein or ligand, a large phenyl ring would result in clashes with Gln730 (Figure 2).

As shown in Table 1, compounds **2**–**9** had improved LE and LLE values compared to hit compound **1**, indicating that potency had been improved in a heavy-atom and lipophilicity efficient manner. Despite the increases in lipophilicity, high aqueous solubility was maintained for all compounds, with a slight reduction for tertiary amide **8**, where a hydrogen bond donor had been removed.

Cell potency was achieved for optimized compounds shown in Table 1, although the differences between enzymatic and cellular potency were not consistent for the compounds. For example, **3** and **7** had 1.7 and 0.7 log unit differences, respectively, despite having the same lipophilicity. These results suggest that lipophilicity-dependent permeability is likely not the sole reason for the differences in biochemical and cellular activity. The ERAP1 gene is polymorphic, resulting in several ERAP1 allotypes being present in human populations, which differ in their enzymatic activity, antigen presentation and sensitivity to allosteric inhibition.^26^ We used the Hap2 construct for the isolated enzyme assay;^9^ however, 74% of the population do not express this haplotype.^26^ Polymorphic variation could contribute to the inconsistent differences in activity between the purified Hap2 construct used in the biochemical assay and the effects on antigen presentation in HeLa cells. Further, the two assays used different peptide substrates; again, this could contribute to inconsistencies in activity between compounds. Despite these considerations, we were encouraged that high potency was achieved in the ERAP1 biochemical and cellular antigen presentation assays for the cyclohexyl acid series.

A variety of point changes of hit compound **1** had resulted in >100-fold increases in potency. Additional X-ray crystal structures were obtained, including chloro compound **2** (see Figure 4). It was clear from the X-ray crystal structure that the lipophilic pocket containing Phe674 and Leu677 was not efficiently filled by the chlorine atom. In our next phase of design, we therefore questioned whether we could increase potency further by filling the lipophilic pocket more efficiently using our structural knowledge. We thus turned to free energy perturbation (FEP) simulations as a tool to prioritize ideas for synthesis.^27^ Several design ideas were predicted using FEP, including a ring contraction of the 6,6-bicyclic motif to a 5,6-bicyclic motif (Figure 3). The hypothesis here was to maintain the hydrogen bond to Gln881, which should act as an anchor, allowing the bicyclic system to rotate and offer a more direct vector to explore the lipophilic pocket.

**Figure 3:**
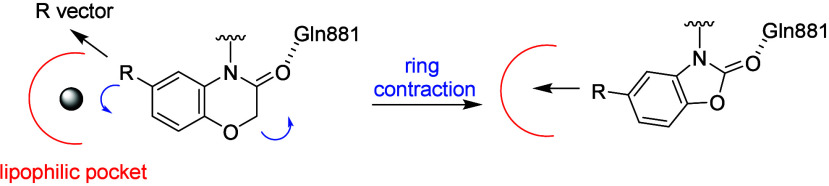
Schematic of the design hypothesis for 5,6-bicyclic systems.

**Figure 4:**
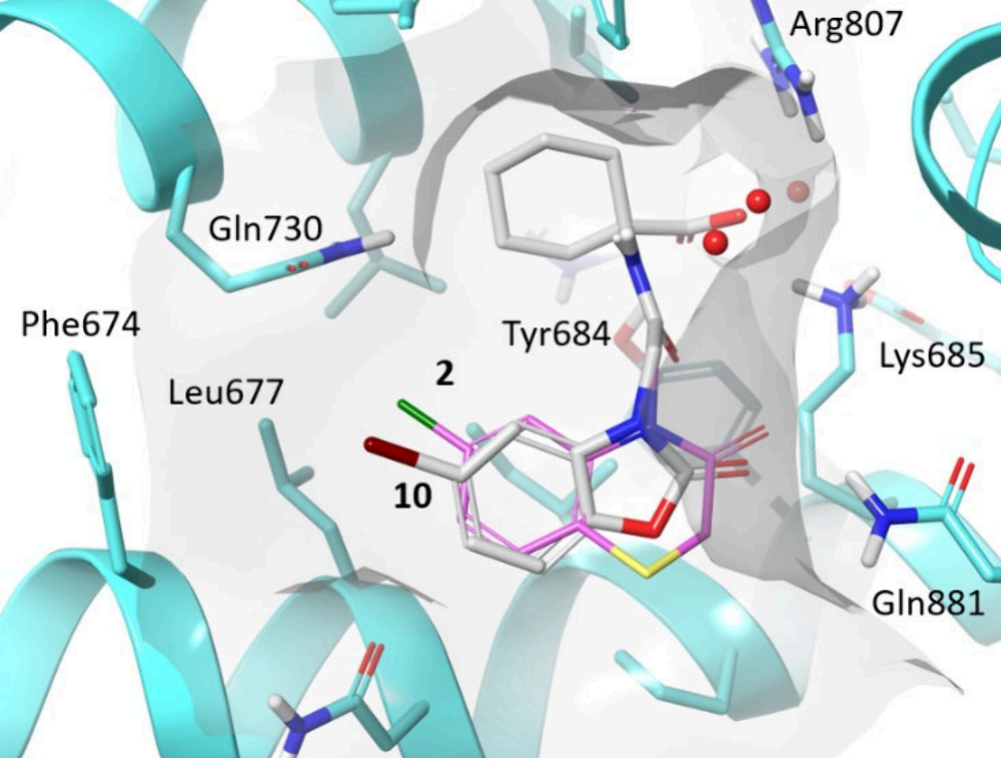
X-ray cocrystal structure of compound **2** (ligand pink, protein blue, resolution 1.33 Å, PDB: 9GK6), overlaid with docked pose of compound **10** (gray) used in FEP simulations.

From the ideas scored and prioritized using binding affinity predictions from the FEP simulations, oxazolidinone **10**, containing a 5,6-bicyclic ring system, had a predicted ERAP1 pIC_50_ of 6.2. When synthesized and tested, this translated into a measured pIC_50_ of 6.6 (Table 2). The binding pose of compound **10** generated for FEP is shown in Figure 4, overlaid with the X-ray crystal structure of compound **2**, which was used as the FEP reference structure. Consistent with our design hypothesis, the structures depicted in Figure 4 suggest a more direct R vector for the Br substituent into the lipophilic pocket compared with the 6,6-bicyclic compound **2**. Based on this modeling, it was thought that the Br atom of compound **10** was not large enough to efficiently fill the lipophilic pocket. Introduction of the larger phenyl motif at R^1^, in compound **11**, resulted in a significantly increased enzymatic pIC_50_ value of 8.1 (Table 2).

**Table 2:**
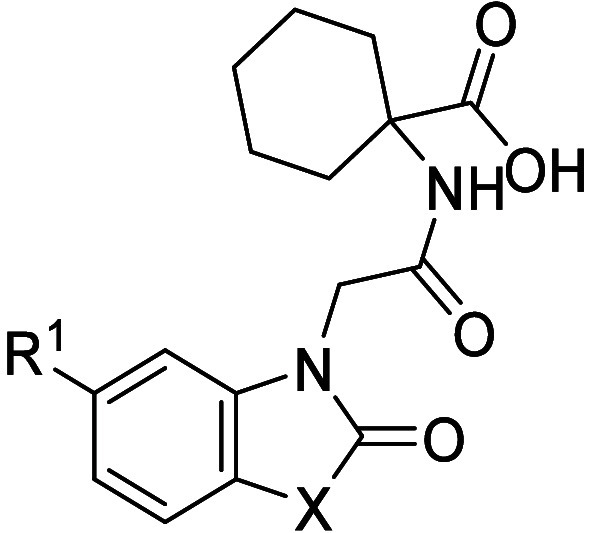

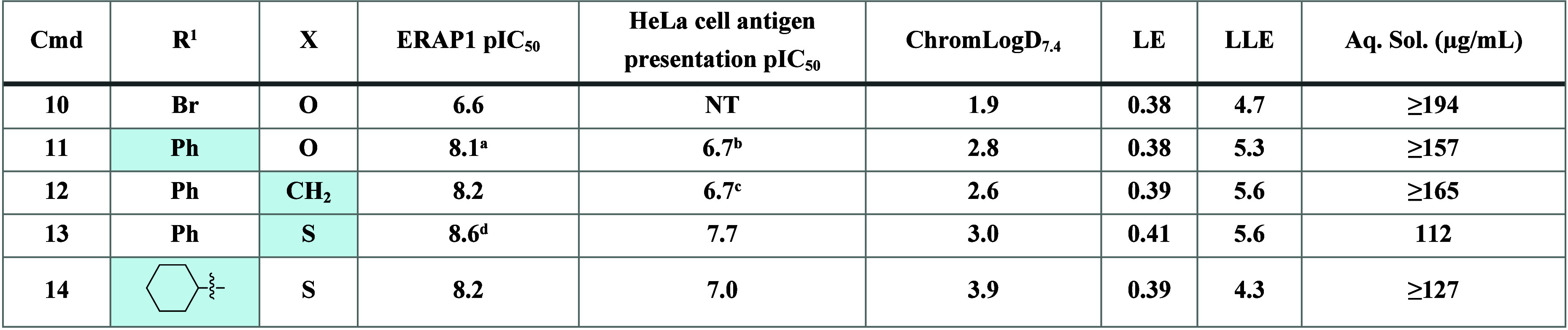
SAR Investigations of 5,6-Bicyclic Systems (ERAP1 and Cell Potency Data Are *n* = 3 or Higher unless Otherwise Stated)

a Tested >8.8 on one test occasion.

b Tested <4.3 on two test occasions.

c *n* = 2.

d Tested >8.8 on two test occasions. NT = not tested.

We then designed further analogues containing 5,6-bicyclic motifs, compounds **12** to **14** (Table 2). Indolinone **12** showed similarly high ERAP1 potency to **11**, and the sulfur variant **13** led to a further increase in enzymatic ERAP1 pIC_50_ to 8.6, and one of the highest LLE values in this set. Nonaromatic alternatives were also well tolerated, as demonstrated by cyclohexyl derivative **14**. With respect to hit compound **1**, the 5,6-bicyclic compounds achieved the objectives of increasing LE and LLE while maintaining high aqueous solubility.

Additional X-ray cocrystal structures of several cyclohexyl acid series compounds were solved, with the aim of understanding the differences in binding between the 6,6- and 5,6-bicyclic systems (Figure 5). The binding modes of compound **7** (ERAP1 pIC_50_ = 7.7) and compound **13** (ERAP1 pIC_50_ = 8.6) largely overlaid well. As predicted for the FEP pose for compound **10**, the most significant difference was in the orientation of the bicyclic ring, which presented the bromine atom and phenyl ring of compounds **7** and **13**, respectively, into the lipophilic pocket. Comparing the two structures, there was also a significant movement of Gln730 to better fit the size and orientation of the ligand. Notably, a range of bicyclic systems, with both small and large substituents, were able to achieve high potency, with significant movement in the pocket observed to accommodate the ligand.

**Figure 5:**
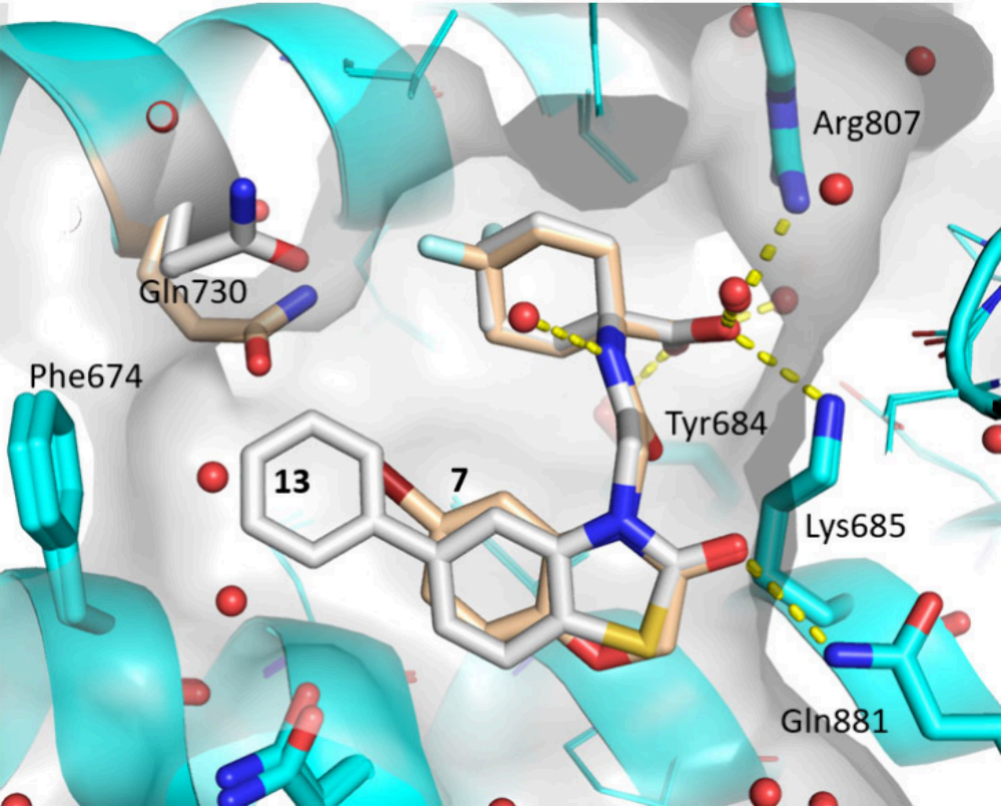
X-ray cocrystal structure overlay of compound **7** (beige, 1.35 Å resolution, PDB: 9GJS) and compound **13** (gray, 1.37 Å resolution, PDB: 9GKE) bound to ERAP1 (cyan). Significant movement in Gln730 was observed between the binding of compounds **7** and **13**.

Both LE and LLE were actively monitored throughout SAR exploration, and these parameters were increased significantly from hit compound **1** (Figure 6). Optimization of LE was pursued by investigating systematic single-point changes without significantly increasing heavy-atom count, for example, in aryl chloride **2**. For LLE, this parameter was optimized by targeting the lipophilic pocket containing Phe674 and Leu677. The objective here was that any increases in potency should be from specific ERAP1 interactions rather than nonspecific lipophilicity-driven binding. These strategies resulted in significant increases in both LE and LLE during optimization. Compound **6** was particularly efficient with the highest LE and LLE values; however, the molecule had significantly lower cellular activity compared to other cyclohexyl acid series compounds.

**Figure 6:**
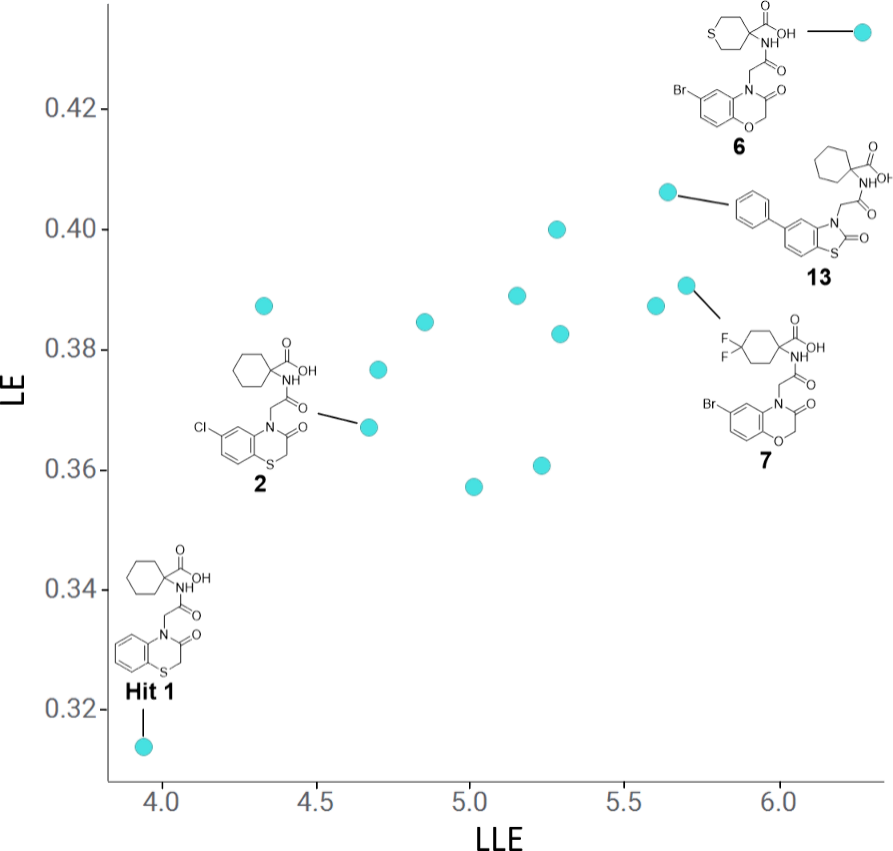
LE and LLE progress from hit **1**.

Given that the cyclohexyl acid series now contained highly potent ERAP1 inhibitors, we sought to understand whether selectivity over ERAP2 and IRAP had been retained from the hit compound **1**. To examine this selectivity, we tested compound **7**, which was one of the most potent compounds in both the biochemical and cellular assays at the time. Selectivity was measured as described previously, by determining pIC_50_ values for hydrolysis of small fluorogenic substrates Arg-AMC or Leu-AMC for ERAP2 and IRAP, respectively.^9^ Pleasingly, **7** showed no measurable inhibition of ERAP2 or IRAP (pIC_50_ < 4.0, *n* = 1) and thus had >1,000-fold ERAP1 selectivity. Selectivity was also observed with the natural product clerodane acid derivative, which was found to bind at the same regulatory site as compound **1** (Figure 1).^9^ Further, docking of structurally related benzofuran compounds, which are selective over ERAP2, indicated binding at the same allosteric site.^12^ This evidence supports the hypothesis that ERAP2 and IRAP do not have the same allosteric regulatory binding site as ERAP1.^28,29^ This binding site hypothesis, in addition to the selectivity data for compounds **1** and **7**, gives us confidence that the potential therapeutic benefits of the cyclohexyl acid series could be tested without potential complications arising from interactions with ERAP2 and IRAP.

Given the progress made in enzymatic and cellular potency for the cyclohexyl acids, we evaluated examples from the series in rat *in vivo* PK studies (Table 3). Compound **13** had a high unbound intravenous clearance and poor oral exposure in rat. However, given the high cellular pIC_50_ value of 7.7, this molecule would potentially be suitable as an *in vitro* tool. In contrast, compound **7** had a significantly reduced unbound *in vivo* clearance with both good oral exposure and bioavailability. Furthermore, compound **7** demonstrated a much lower *in vitro* intrinsic clearance in rat and human hepatocytes, is 10-fold less lipophilic than compound **13**, and has a much higher FaSSIF solubility. Taken together, this may explain, in part, the difference in oral bioavailability between compound **7** and compound **13**. Following the expected lipophilicity trend,^27^ the more polar compound **7** had a higher fraction unbound value in rat blood. The volume of distribution for both compounds was surprisingly high, where carboxylic acids typically have volume of distribution values between 0.1 and 0.4 L/kg.^30^ Both compounds had moderate permeabilities in the Caco-2 permeability assay. Given the encouraging *in vivo* PK results of compound **7**, the cyclohexyl acid series was considered a promising series for lead optimization.

**Table 3:** *In Vitro* Clearance and *In Vivo* Rat PK Data for Compounds **7** and **13**

		IV			PO				*In vitro* Hepatocyte CL_int_(mL/min/g tissue)			
Cmd	Dose IV/PO (mg/kg)	*V*_d,ss_ (L/kg)	CL/CL_u_^a^ (mL/min/kg)	*T*_1/2_(h)	*C*_max_ (ng/mL)	AUC_0-*t*_ (ng·h/mL)	Rat *F*_u,blood_	%*F*	Human	Rat	FaSSIF sol. 4 h (μg/mL)	Caco-2 Papp (AP/BL) (nm/s)
**7**	1.0/2.6	4.0	39.0/283	3.6	67.1	229	0.138	18	0.52	0.63	>1,000	48
**13**	1.0/2.0	2.1	79.6/7236	0.98	8.0	10.0	0.011	3	1.97	5.44	58	30

a Cl_u_ = unbound *in vivo* clearance, calculated by dividing intravenous clearance (IV CL) by *F*_u,blood_.

In conclusion, a series of ERAP1 inhibitors have been developed, examples of which have >1,000-fold increased potency over hit cyclohexyl acid **1**. High potency in the HeLa cell antigen presentation assay was also achieved, with the most potent example, **13**, displaying a pIC_50_ of 7.7. Optimization focused on a combination of systematic single-atom point changes and structure-based drug design supported by FEP potency predictions. X-ray crystallography confirmed that the 5,6-bicyclic motifs accessed the lipophilic pocket formed by Phe674 and Leu677 more directly compared to the 6,6-bicyclic systems. As part of the optimization, active pursuit and tracking of LE and LLE efficiency metrics resulted in simultaneous increases in both of these parameters. Both hit compound **1** and lead compound **7** were found to be selective over ERAP2 and IRAP. Furthermore, compound **7** had promising pharmacokinetic properties in rats, providing an excellent starting point for further optimization, which will be the subject of future publications.

## Safety Statement

No unexpected safety observations were noted from this work. Novel compounds were assumed toxic as a precaution.

## Data Availability

The coordinates and structure factors have been deposited in the Protein Data Bank under the accession codes 9GJN (**1**), 9GK6 (**2**), 9GJS (**7**), and 9GKE (**13**).

## Supporting Information Available

Compound synthesis and characterization, LCMS and NMR traces for key compounds, assay standard deviations and replicates, assay methods, X-ray crystallography methods, FEP methods (PDF)

## Notes

Human biological samples were sourced ethically, and their research use was in accord with the terms of the informed consents under an IRB/REC approved protocol. All animal studies were ethically reviewed and carried out in accordance with Animals (Scientific Procedures) Act 1986 and the GSK Policy on the Care, Welfare, and Treatment of Animals.

## Abbreviations

ADMET: absorption, distribution, metabolism, excretion, toxicity
CAD: charged aerosol detection
ERAP2: endoplasmic reticulum amino peptidase 2
FEP: free energy perturbation
IRAP: insulin-regulated aminopeptidase
LE: ligand efficiency
LLE: lipophilic ligand efficiency
MHC-I: major histocompatibility class I
PK: pharmacokinetics
SAR: structure–activity relationship
