# Supplementary material for: Optimization of Potent and Selective Cyclohexyl Acid ERAP1 Inhibitors Using Structure- and Property-Based Drug Design
Source: ACS Med Chem Lett. 2024 Nov 6;15(12):2107–14. doi: 10.1021/acsmedchemlett.4c00401 (PMC11647717; doi:10.1021/acsmedchemlett.4c00401)
Supplement: Supplementary file 1 — ml4c00401_si_001.pdf [file ml4c00401_si_001.pdf]

Optimization of Potent and Selective Cyclohexyl Acid ERAP1 Inhibitors Using Structure- and Property-Based Drug Design

Ross P. Hryczanek*,[#,‡], Andrew S. Hackett[#], Paul Rowland[#], Chun-wa Chung[#], Máire A. Convery[#], Duncan S. Holmes[#], Jonathan P. Hutchinson[#], Semra Kitchen[#], Justyna Korczynska[#], Robert P. Law[#], Jonathan D. Lea[#], John Liddle[#], Richard Lonsdale[#], Margarete Neu[#], Leng Nickels[#], Alex Phillipou[#], James E. Rowedder[#], Jessica L. Schneck[#], Paul Scott-Stevens[#], Hester Sheehan[#], Chloe L. Tayler[#], Ioannis Temponeras[†], Christopher P. Tinworth[#], Ann L. Walker[#], Justyna Wojno-Picon[#], Robert J. Young[#], David M. Lindsay[‡] and Efstratios Stratikos[†]

[#]GSK, Medicines Research Centre, Gunnels Wood Road, Stevenage, SG1 2NY, U.K.
[†]National Center for Scientific Research "Demokritos", Agia Paraskevi, Attiki, 15341, Greece
[‡]Department of Pure and Applied Chemistry, University of Strathclyde, Glasgow G1 1XL, U.K.

Supporting Information

Contents

General Chemistry Procedures..2
Synthetic Procedures..4
Experimental Procedures..6
LCMS and NMR Traces for Key Compounds..30
Cellular Antigen Presentation Assay..34
Assay Standard Deviations and Replicates..35
X-Ray Crystallography Methods..37
FEP Methods..40
DMPK Methods..41
Physicochemical Property Data..44
References..45

General Chemistry Procedures

All compounds used for biological testing were >95% pure by HPLC or LCMS.

Unless otherwise stated, all reactions were carried using anhydrous solvents. Solvents and reagents were purchased from commercial suppliers and used as received. Reactions were monitored by thin layer chromatography (TLC) or LCMS. TLC was carried out on glass or aluminium-backed 60 silica plates coated with UV254 fluorescent indicator. Spots were visualised using UV light (254 or 365 nm) or common staining methods as appropriate. Silica flash chromatography was carried out using Teledyne Isco CombiFlash® apparatus using RediSep® pre-packed silica cartridges. Extracted organic mixtures were dried using Biotage PTFE hydrophobic phase separator frits unless otherwise stated.

NMR spectra were recorded at rt (unless otherwise stated) using standard pulse methods on a Bruker AV-400 spectrometer (^{1}H = 400 MHz, ^{13}C = 101 MHz). Chemical shifts are referenced to trimethylsilane (TMS) or the residual solvent peak, and are reported in ppm. Coupling constants are reported in Hz and refer to $^{3}J_{H\text{-}H}$ couplings, unless otherwise stated. Coupling constants are quoted to the nearest 0.1 Hz and multiplicities are given by the following abbreviations and combinations thereof: s (singlet), d (doublet), ABq (AB quartet), t (triplet), q (quartet), quin (quintet), sxt (sextet), m (multiplet), br. (broad). Pairs of coupling constants were averaged to the nearest 0.1 Hz.

IR spectra were obtained on a Perkin Elmer Spectrum 1 FTIR apparatus, with major peaks reported (cm^{-1}). HRMS data was recorded on a Micromass Q-Tof Ultima hybrid quadrupole time-of-flight mass spectrometer, with analytes separated on an Agilent 1100 Liquid Chromatograph equipped with a Phenomenex Luna C18(2) reversed phase column (100 mm x 2.1 mm, 3 µm packing diameter). LC conditions were 0.5 mL/min flow rate, 35 ºC, injection volume 2-5 µL. Gradient elution with (A) water containing 0.1% (v/v) formic acid and (B) acetonitrile containing 0.1% (v/v) formic acid. Gradient conditions were initially 5% B, increasing linearly to 100% B over 6 min, remaining at 100% B for 2.5 min then decreasing linearly to 5% B over 1 min followed by an equilibration period of 2.5 min prior to the next injection.

LCMS analysis was carried out on a Waters Acquity UPLC instrument equipped with a BEH or CSH column (50 mm x 2.1 mm, 1.7 µm packing diameter) and Waters micromass ZQ MS using alternate-scan positive and negative electrospray. Analytes were detected as a summed UV wavelength of 210 – 350 nm. Two liquid phase methods were used:

Formic: 40 °C, 1 mL/min flow rate. Gradient elution with the mobile phases as (A) water containing 0.1% volume/volume (v/v) formic acid and (B) acetonitrile containing 0.1% (v/v) formic acid. Gradient conditions were initially 1% B, increasing linearly to 97% B over 1.5 min, remaining at 97% B for 0.4 min then increasing to 100% B over 0.1 min.

High pH: 40 °C, 1 mL/min flow rate. Gradient elution with the mobile phases as (A) 10 mM aqueous ammonium bicarbonate solution, adjusted to pH 10 with 0.88 M aqueous ammonia and (B) acetonitrile. Gradient conditions were initially 1% B, increasing linearly to 97% B over 1.5 min, remaining at 97% B for 0.4 min then increasing to 100% B over 0.1 min.

Mass directed automatic purification (MDAP)

Formic MDAP: The HPLC separation was conducted on an Xselect CSH C18 column (150 mm x 30 mm i.d. 5 µm packing diameter) at ambient temperature, eluting with 0.1% formic acid in water (solvent A) and 0.1% formic acid in acetonitrile (solvent B) using an elution gradient of between 0 and 100% solvent B over 15 or 25 min. The UV detection was an averaged signal from wavelength of 210 nm to 350 nm. The mass spectra were recorded on a Waters ZQ Mass Spectrometer using alternate-scan positive and negative electrospray.

High pH MDAP: The HPLC analysis was conducted on an Xselect CSH C18 column (150 mm x 30 mm i.d. 5 µm packing diameter) at ambient temperature, eluting with 10 mM ammonium bicarbonate in water adjusted to pH 10 with ammonia solution (solvent A) and acetonitrile (solvent B) using an elution gradient of between 0 and 100% solvent B over 15 or 25 min. The UV detection was an averaged signal from wavelength of 210 nm to 350 nm. The mass spectra were recorded on a Waters ZQ Mass Spectrometer using alternate-scan positive and negative electrospray.

Synthetic Procedures

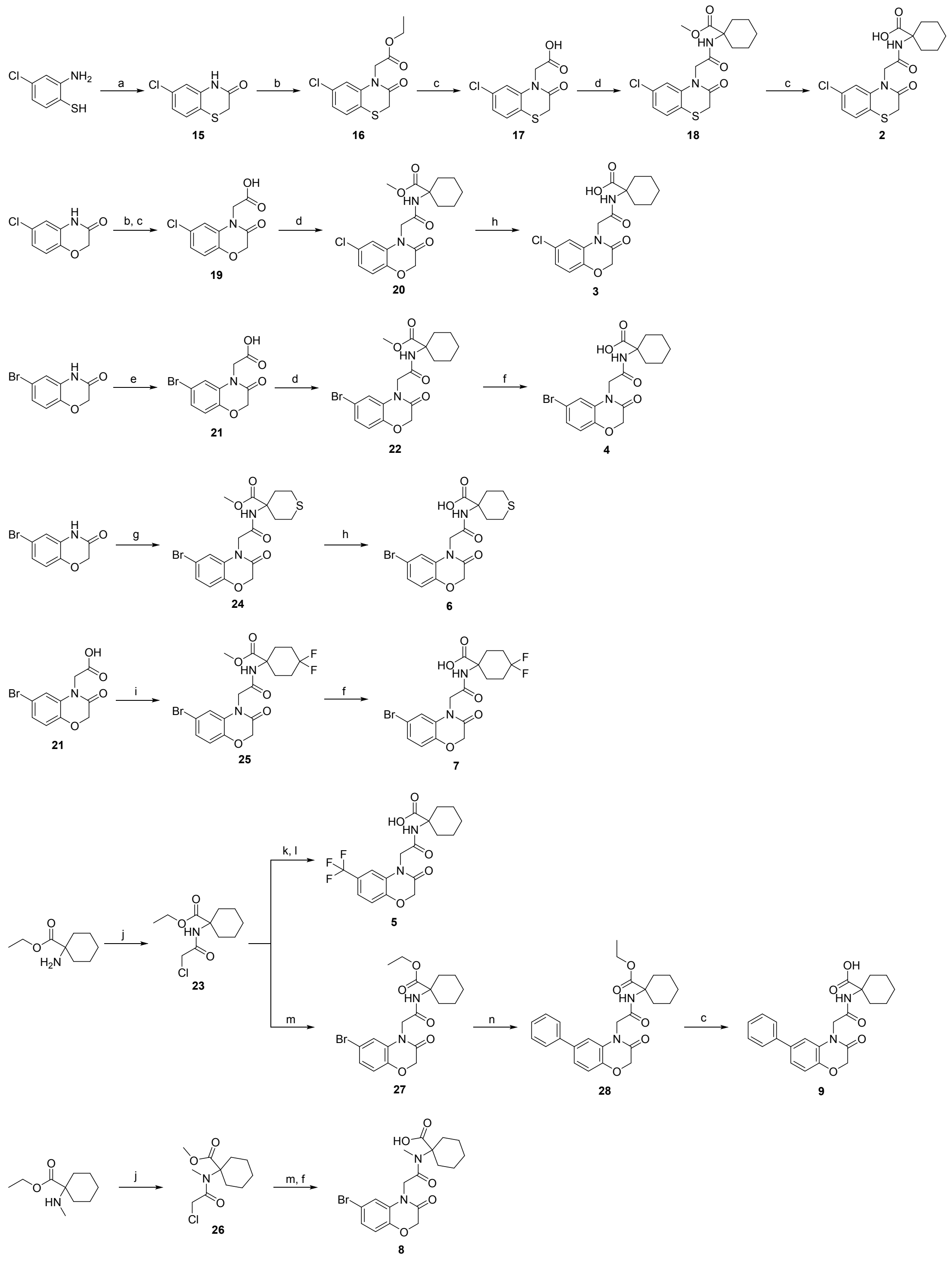

Scheme S1. Synthesis of 6,6 core compounds. a) chloroacetyl chloride, K_3PO_4, DMF, 60 °C; b) ethyl bromoacetate, NaI, K_2CO_3, EtOH, 60 or 80 °C; c) LiOH, EtOH or THF, water, rt or 60 °C; d) methyl 1-aminocyclohexane-1-carboxylate, HATU, DIPEA, DMF, rt or 0 °C; e) ethyl bromoacetate, K_2CO_3, EtOH, 60 °C, then LiOH; f) LiOH, MeOH, water, 60 °C; g) i. methyl 4-aminotetrahydro-2*H*-thiopyran-4-carboxylate, chloroacetyl chloride, Et_3N, DCM, 0 °C, ii. 6-

bromo-2*H*-benzo[*b*][1,4]oxazin-3(4*H*)-one, 18-crown-6, K_2CO_3, MeCN, 60 °C; h) HCl, 1,4-dioxane, water, 0 °C or 60 °C; i) methyl 1-amino-4,4-difluorocyclohexane-1-carboxylate, HATU, DIPEA, DMF, rt; j) Et_3N, chloroacetyl chloride, DCM, 0 °C or rt; k) 6-(trifluoromethyl)-2H-benzo[*b*][1,4]oxazin-3(4*H*)-one, K_2CO_3, 18-crown-6, MeCN, 60 °C; l) LiOH, EtOH, water, 60 °C; m) 6-bromo-2*H*-benzo[*b*][1,4]oxazin-3(4*H*)-one, 18-crown-6, K_2CO_3, MeCN, 60 °C; n) PhBPin, K_2CO_3, $PdCl_2(PPh_3)_2$, DMF, water, 100 °C.

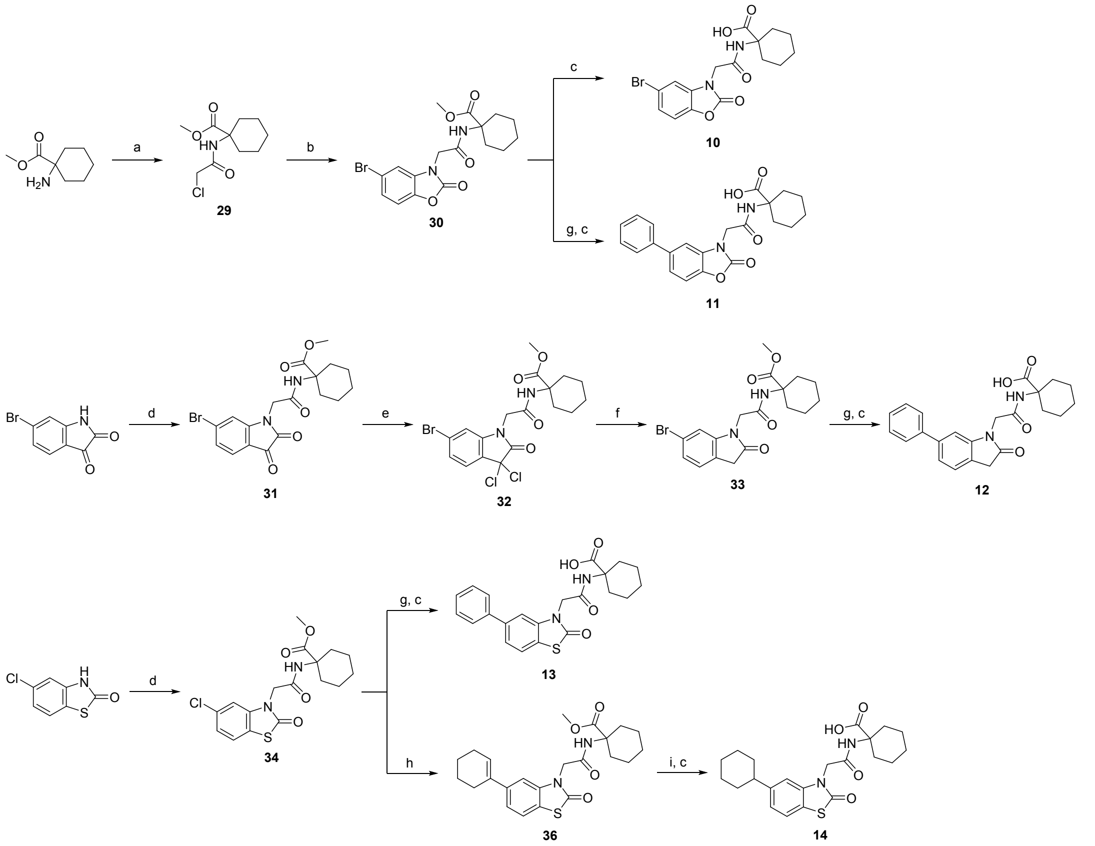

Scheme S2. Synthesis of 5,6-core compounds. a) chloroacetyl chloride, Et_3N, DCM, 0 °C; b) 5-bromobenzo[*d*]oxazol-2(3*H*)-one, K_2CO_3, 18-crown-6, MeCN, 60 °C; c) HCl, 1,4-dioxane, water, 60 °C; d) **29**, K_2CO_3, 18-crown-6, MeCN, 60 °C; e) PCl_5, toluene, 60 °C; f) Zn, AcOH, rt; g) PhBPin, K_2CO_3, Pd(amphos)Cl_2, 1,4-dioxane, water, 80 °C; h) cyclohex-1-ene boronic acid, Pd(amphos)Cl_2, K_2CO_3, 1,4-dioxane, water, 80 °C; i) H_2, Pd/C, EtOH, 40 °C.

Experimental Procedures

1-(2-(3-oxo-2,3-dihydro-4*H*-benzo[*b*][1,4]thiazin-4-yl)acetamido)cyclohexane-1-carboxylic acid (1)

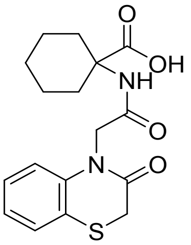

Purchased from commercial vendor.

6-Chloro-2*H*-benzo[*b*][1,4]thiazin-3(4*H*)-one (15)

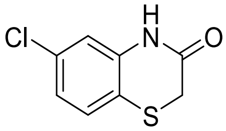

To a solution of 2-amino-4-chlorobenzenethiol (2.0 g, 12.53 mmol) in DMF (10 mL) was added K_2CO_3 (5.1 g, 37.39 mmol, and chloroacetyl chloride (1.0 mL, 12.53 mmol) under nitrogen. The reaction was stirred at 60 °C for 1 h. The reaction was quenched with ice water (30 mL) and extracted with EtOAc (2 x 20 mL), organics were washed with brine (2 x 15 mL), dried using sodium sulfate and evaporated. Purified by silica chromatography (50g SNAP cartridge, 0-50% EtOAc/hexane). Fractions were evaporated to afford to give 6-chloro-2*H*-benzo[*b*][1,4]thiazin-3(4*H*)-one **15** (1 g, 5.02 mmol, 42% yield) as a brown gummy solid. LCMS (formic) t_R = 1.83 min, *m/z* $[M + H]^+$ = 200; ^1H NMR (400 MHz, DMSO-d_6) δ 10.69 (s, 1H), 7.34 (d, *J* = 8.0 Hz, 1 H), 7.04-7.00 (m, 2H), 3.49 (s, 2H).

Ethyl 2-(6-chloro-3-oxo-2,3-dihydro-4*H*-benzo[*b*][1,4]thiazin-4-yl)acetate (16)

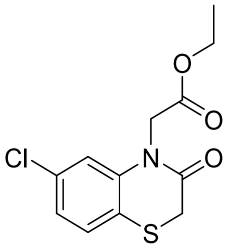

To a solution of 6-chloro-2*H*-benzo[*b*][1,4]thiazin-3(4*H*)-one **15** (1 g, 5.02 mmol) in ethanol (10 mL) was added K_2CO_3 (2.0 g, 15.05 mmol), ethyl bromoacetate (0.66 mL, 12.53 mmol)

and sodium iodide (735 mg, 5.02 mmol) under nitrogen. The reaction was stirred at rt for 10 min, then heated to 80 °C for 16 h. The reaction was concentrated under reduced pressure then was quenched with ice water (15 mL) and extracted with EtOAc (2 x 20 mL), organics were washed with brine (2 x 10 mL), dried using sodium sulfate and evaporated. Purified by silica chromatography (25g SNAP cartridge, 0-50% EtOAc/petroleum ether). Fractions were evaporated to afford ethyl 2-(6-chloro-3-oxo-2,3-dihydro-4*H*-benzo[*b*][1,4]thiazin-4-yl)acetate **16** (408 mg, 1.43 mmol, 29%) as a pale yellow solid. LCMS (formic) t_R = 2.19 min, *m/z* $[M + H]^+$ = 286; ^{1}H NMR (400 MHz, DMSO-d_6) δ 7.47 (d, *J* = 8.0 Hz, 1 H), 7.29 (d, *J* = 2 Hz, 1H), 7.15 (dd, *J* = 8.0, 2.0 Hz, 1H), 4.74 (s, 2H), 4.15 (q, *J* = 5.2 Hz, 2H), 3.60 (s, 2H), 1.21 (t, *J* = 5.2 Hz, 3H).

2-(6-Chloro-3-oxo-2,3-dihydro-4*H*-benzo[*b*][1,4]thiazin-4-yl)acetic acid (17)

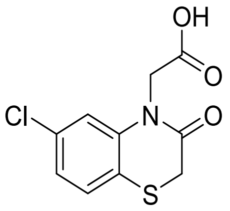

To a solution of ethyl 2-(6-chloro-3-oxo-2,3-dihydro-4*H*-benzo[*b*][1,4]thiazin-4-yl)acetate **16** (400 mg, 1.4 mmol) in THF (5 mL) and water (5 mL) was added $LiOH.H_2O$ (234 mg, 5.60 mmol). The reaction was stirred at rt for 4 h. The reaction concentrated under reduced pressure then was quenched with ice water (5 mL), then slowly acidified with citric acid solution until a precipitate formed. The precipitate was collected by filtration and washed with water and diethyl ether, then dried under reduced pressure to give 2-(6-chloro-3-oxo-2,3-dihydro-4*H*-benzo[*b*][1,4]thiazin-4-yl)acetic acid **17** (300 mg, 1.16 mmol, 83% yield) as an off-white solid. LCMS (formic) t_R = 1.73 min, *m/z* $[M + H]^+$ = 276.

Methyl 1-(2-(6-chloro-3-oxo-2,3-dihydro-4*H*-benzo[*b*][1,4]thiazin-4-yl)acetamido)cyclohexane-1-carboxylate (18)

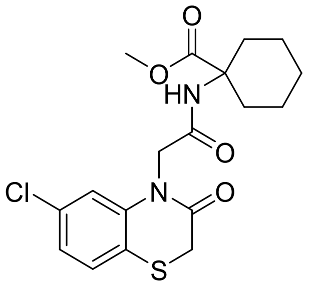

To a solution of 2-(6-chloro-3-oxo-2,3-dihydro-4*H*-benzo[*b*][1,4]thiazin-4-yl)acetic acid **17** (750 mg, 2.91 mmol) in DMF (5 mL) at 0 °C was added DIPEA (2.1 mL, 11.64 mmol) and HATU (1.6 g, 4.37 mmol). Methyl 1-aminocyclohexane-1-carboxylate (457 mg, 2.91 mmol) was added and the reaction was stirred at rt for 4 h. To the reaction was added ice water (30 mL) and extracted with EtOAc (2 x 15 mL), organics were washed with brine (2 x 10 mL), dried using sodium sulfate and evaporated. Purified by silica chromatography (25g SNAP cartridge, 0-80% EtOAc/petroleum ether). Fractions were evaporated to afford to give methyl 1-(2-(6-chloro-3-oxo-2,3-dihydro-4*H*-benzo[*b*][1,4]thiazin-4-yl)acetamido)cyclohexane-1-carboxylate **18** (600 mg, 1.51 mmol, 54%) as a pale yellow solid. LCMS (formic) t_R = 2.19 min, *m/z* $[M + H]^+$ = 397, 88% purity; ^1H NMR (400 MHz, DMSO-d_6) δ 8.39 (s, 1H), 7.44 (d, *J* = 8.0 Hz, 1 H), 7.13 (dd, *J* = 8.0, 2.0 Hz, 1H), 6.99 (d, *J* = 2 Hz, 1H), 4.62 (s, 2H), 3.63-3.55 (m, 5H), 1.97-1.22 (m, 10H).

1-(2-(6-Chloro-3-oxo-2,3-dihydro-4*H*-benzo[*b*][1,4]thiazin-4-yl)acetamido)cyclohexane-1-carboxylic acid (2)

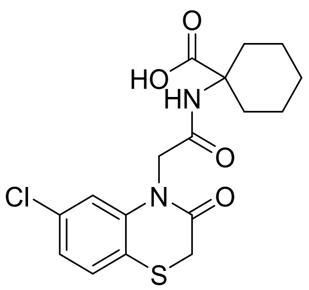

To a solution of methyl 1-(2-(6-chloro-3-oxo-2,3-dihydro-4*H*-benzo[*b*][1,4]thiazin-4-yl)acetamido)cyclohexane-1-carboxylate **18** (300 mg, 0.73 mmol) in THF (4 mL) and water (4 mL) at 0 °C was added $LiOH.H_2O$ (127 mg, 3.03 mmol). The reaction was stirred at rt for 4 h. The reaction was concentrated under reduced pressure then was diluted with ice water (5 mL), then cooled to 0 °C and acidified with citric acid solution (10 mL). The aqueous was extracted

with 10% MeOH in DCM and dried over sodium sulfate, then concentrated under vacuum. Purified by silica chromatography (25g SNAP cartridge, 0-3% MeOH/DCM). Fractions were evaporated to afford to give 1-(2-(6-chloro-3-oxo-2,3-dihydro-4*H*-benzo[*b*][1,4]thiazin-4-yl)acetamido)cyclohexane-1-carboxylic acid **2** (40mg, 0.10 mmol, 14% yield) as an off-white solid. LCMS (formic$^+$) t_R = 1.97 min, *m/z* $[M + H]^+$ = 383; HPLC (TFA): t_R = 7.77 min, 95.7 % purity; ^1H NMR (400 MHz, DMSO-d_6) δ 12.26 (s, 1H), 8.20 (s, 1H), 7.44 (d, *J* = 8.0 Hz, 1 H), 7.07-7.06 (m, 2H), 4.61 (s, 2H), 3.56 (s, 2H), 1.99-1.98 (m, 2H), 1.68-1.59 (m, 7H), 1.47-1.24 (m, 1H).

2-(6-Chloro-3-oxo-2,3-dihydro-4*H*-benzo[*b*][1,4]oxazin-4-yl)acetic acid (19)

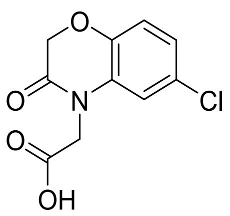

6-Chloro-2*H*-benzo[*b*][1,4]oxazin-3(4*H*)-one (1.0 g, 5.5 mmol) in ethanol (30 mL) was treated with K_2CO_3 (1.88 g, 13.62 mmol) followed by ethyl 2-bromoacetate (0.664 mL, 5.99 mmol) and the reaction stirred at 60 °C under a nitrogen atmosphere for 17.5 h. The reaction mixture was cooled to rt, then treated with ethyl 2-bromoacetate (0.181 mL, 1.63 mmol) and stirred at 60 °C under a nitrogen atmosphere for 1 h. The reaction mixture was treated with LiOH hydrate (0.457 g, 10.9 mmol) and water (10 mL) and stirred at 60 °C for 5.5 h. The reaction mixture was concentrated under reduced pressure. The residue was treated with water (50 mL) and DCM (100 mL) and the aqueous phase isolated. The aqueous phase was acidified to ~pH 3 carefully by treatment with 25% aqueous HCl dropwise and extracted with DCM (2 × 100 mL). The combined organic phases were passed through a hydrophobic frit and the solvent removed under reduced pressure to yield 2-(6-chloro-3-oxo-2,3-dihydro-4*H*-benzo[*b*][1,4]oxazin-4-yl)acetic acid **19** (1.19 g, 4.92 mmol, 90% yield) as a white solid. LCMS (formic) t_R = 0.80 min, *m/z* $[M + H]^+$ = 242; IR ν_{max} (cm^{-1}) 2961, 1730, 1651, 1601, 1588, 1498, 1454; ^1H NMR (400 MHz, DMSO-d_6) δ 13.09 (br. s, 1H), 7.22-7.18 (m, 1H), 7.08-7.03 (m, 2H), 4.71 (s, 2H), 4.66 (s, 2H); ^{13}C NMR (101 MHz, DMSO-d_6) δ 169.1, 164.2, 143.5, 130.1, 126.4, 123.1, 117.9, 115.2, 66.8, 42.5; HRMS ($C_{10}H_9ClNO_4$): $[M + H]^+$ calculated 242.0220, found 242.0218

Methyl 1-(2-(6-chloro-3-oxo-2,3-dihydro-4*H*-benzo[*b*][1,4]oxazin-4-yl)acetamido)cyclohexane-1-carboxylate (20)

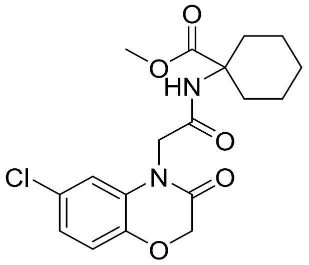

To a solution of 2-(6-chloro-3-oxo-2,3-dihydro-4*H*-benzo[*b*][1,4]oxazin-4-yl)acetic acid **19** (1.5 g, 6.21 mmol) in DMF (20 mL), was added HATU (3.54 g, 9.31 mmol) and DIPEA (4.34 mL, 24.83 mmol) at 0 °C and stirred for 15 min. To this was added methyl 1-aminocyclohexane-1-carboxylate (0.976 g, 6.21 mmol) portionwise and the stirring continued for 3 h. The reaction mixture was poured into ice water (50 mL) and was extracted with EtOAc (20 mL x 3). The organic layer was separated and washed with brine solution (20 mL). The EtOAc portion was dried over anhydrous sodium sulphate (5 g), then filtered and concentrated under reduced pressure to yield the crude product. The crude was adsorbed onto silica gel and purified by normal phase chromatography (50g SNAP silica column, 10 % EtOAc in petroleum ether). The combined eluents were concentrated under reduced pressure to obtain methyl 1-(2-(6-chloro-3-oxo-2,3-dihydro-4*H*-benzo[*b*][1,4]oxazin-4-yl)acetamido)cyclohexane-1-carboxylate **20** (1.51 g, 3.34 mmol, 53.9 % yield) as a yellow solid. LCMS (ES^+) t_R = 2.109 min, *m/z* $[M + H]^+$ = 381; ^{1}H NMR (400 MHz, DMSO-d_6) δ 8.47 (s, 1H), 7.08-7.03 (m, 2H), 6.97 (d, *J* = 2 Hz, 1H), 4.72 (s, 2H), 4.61 (s, 2H), 3.51 (s, 3H), 2.09 – 1.23 (m, 10H).

1-(2-(6-Chloro-3-oxo-2,3-dihydro-4*H*-benzo[*b*][1,4]oxazin-4-yl)acetamido)cyclohexane-1-carboxylic acid (3)

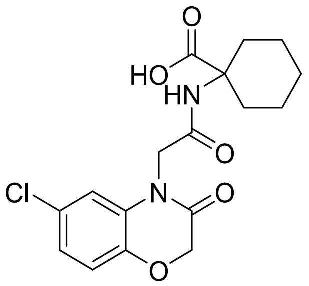

To a solution of methyl 1-(2-(6-chloro-3-oxo-2,3-dihydro-4*H*-benzo[*b*][1,4]oxazin-4-yl)acetamido)cyclohexane-1-carboxylate **20** (500 mg, 1.31 mmol) in 1,4-dioxane (20 mL) was added 1.5 M aqueous HCl (1.75 mL, 2.63 mmol) at 0 °C and stirred for 2 h. The reaction

mixture was evaporated under vacuum, basified with saturated Na_2CO_3 solution and extracted with EtOAc (2 x 20 mL). The aqueous layer was separated and the organic layer was discarded. This aqueous portion was again acidified with 1.5 N HCl to pH = 4-5. This was again extracted with EtOAc (2 x 20 mL) and the combined organic layers were separated and dried over anhydrous sodium sulphate (5 g) and filtered. The filtrate was evaporated to dryness to give the crude material as an off white solid. Purified by preparative HPLC (Sunfire C18 (19x150mm) 5μm) using a 0.1% aqueous formic acid/acetonitrile gradient. Fractions were dried, co-distilled with 10 % MeOH in DCM (3 x 15 mL) and dried under vacuum to afford 1-(2-(6-chloro-3-oxo-2,3-dihydro-4*H*-benzo[*b*][1,4]oxazin-4-yl)acetamido)cyclohexane-1-carboxylic acid **3** (70 mg, 0.19 mmol, 14 % yield), as a white solid. LCMS (ES^+) t_R = 1.904 min, *m/z* $[M + H]^+$ = 367, 99.5% purity; HPLC: t_R: 7.047 min, 99.9% purity; ^1H NMR (400 MHz, DMSO-d_6) δ 12.29 (s, 1H), 8.22 (s, 1H), 7.03-7.01 (m, 3H), 4.70 (s, 2H), 4.57 (s, 2H), 2.02 (d, J = 12.40 Hz, 2H), 2.04-2.01 (m, 7H), 1.24 (s, 1H).

2-(6-Bromo-3-oxo-2,3-dihydro-4*H*-benzo[*b*][1,4]oxazin-4-yl)acetic acid (21)

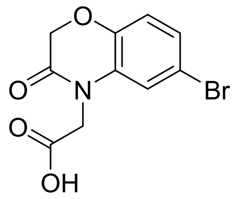

6-bromo-2*H*-benzo[*b*][1,4]oxazin-3(4*H*)-one (5.0 g, 22 mmol) in ethanol (150 mL) was treated with K_2CO_3 (7.6 g, 55 mmol) and ethyl 2-bromoacetate (3.39 mL, 30.5 mmol) and the reaction stirred under a nitrogen atmosphere at 60 °C for 18 h. The reaction mixture was treated with LiOH hydrate (2.8 g, 67 mmol) and stirred at 60 °C for 1 h. The solvent was then removed under reduced pressure. The residue was treated with water (1 L) and DCM (500 mL). The aqueous phase was brought to pH 1 by dropwise addition of aqueous HCl (2 M). The aqueous phase was extracted with DCM (3 L) and the solvent removed under reduced pressure to yield 2-(6-bromo-3-oxo-2,3-dihydro-4*H*-benzo[*b*][1,4]oxazin-4-yl)acetic acid **21** (4.04 g, 14.1 mmol, 64% yield, 85% purity) as a white solid. LCMS (formic) t_R = 0.82 min, *m/z* $[M + H]^+$ = 286; ^1H NMR (400 MHz, DMSO-d_6) δ 13.11 (br. s, 1H), 7.29 (d, ^4J = 2.0 Hz, 1H), 7.18 (dd, ^4J = 2.0, J = 8.4 Hz, 1H), 6.99 (d, J = 8.4 Hz, 1H), 4.71 (s, 2H), 4.65 (s, 2H)

Methyl 1-(2-(6-bromo-3-oxo-2,3-dihydro-4*H*-benzo[*b*][1,4]oxazin-4yl)acetamido)cyclohexane-1-carboxylate (22)

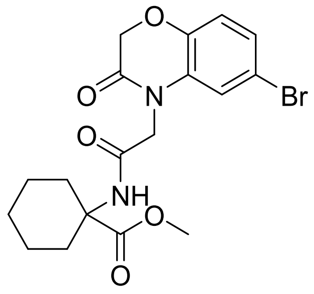

2-(6-bromo-3-oxo-2,3-dihydro-4*H*-benzo[*b*][1,4]oxazin-4-yl)acetic acid **21** (2.75g, 9.61 mmol) was treated with methyl 1-aminocyclohexane-1-carboxylate (1.693 g, 10.77 mmol), DIPEA (5.04 mL, 28.8 mmol), HATU (4.39 g, 11.5 mmol) and DMF (30 mL) then the reaction stirred at rt for 24 h. The reaction mixture was treated with HATU (2.19 g, 5.77 mmol) and stirred at rt for 1 h. The solvent was removed under reduced pressure. The residue was treated with aqueous saturated $NaHCO_3$ (20 mL) and DCM (100 mL) and the organic phase isolated by filtration through a hydrophobic frit. The organic phase was treated with aqueous HCl (1 M) then the organic phase isolated by filtration through a hydrophobic frit. The solvent was then removed under reduced pressure. The residue was purified by Si column chromatography (0-50% EtOAc/cyclohexane) to yield methyl 1-(2-(6-bromo-3-oxo-2,3-dihydro-4*H*-benzo[*b*][1,4]oxazin-4yl)acetamido)cyclohexane-1-carboxylate **22** (1.77 g, 4.16 mmol, 43%). LCMS (formic) t_R = 1.06 min, *m/z* $[M + H]^+$ = 425. IR ν_{max} (cm^{-1}) 3288, 2939, 2857, 1736, 1697, 1677, 1651, 1601; ^1H NMR (400 MHz, DMSO-d_6) δ 8.43 (s, 1H), 7.22-7.14 (m, 1H), 7.05 (d, ^4J = 2.5 Hz, 1H), 6.97 (d, J = 8.4 Hz, 1H), 4.71 (s, 2H), 4.60 (s, 2H), 3.52 (s, 3H), 2.00-1.89 (m, 2H), 1.73-1.61 (m, 2H), 1.60-1.46 (m, 5H), 1.35-1.20 (m, 1H); ^{13}C NMR (101 MHz, DMSO-d_6) δ 174.5, 166.4, 164.4, 144.4, 130.9, 126.4, 118.7, 118.3, 114.3, 67.3, 58.8, 52.2, 43.2, 32.2, 25.3, 21.4; HRMS ($C_{18}H_{22}{}^{79}BrN_2O_5$): $[M + H]^+$ calculated 425.0712, found 425.0710

1-(2-(6-Bromo-3-oxo-2,3-dihydro-4*H*-benzo[*b*][1,4]oxazin-4yl)acetamido)cyclohexane-1-carboxylic acid (4)

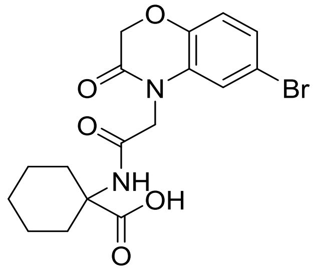

A solution of methyl 1-(2-(6-bromo-3-oxo-2,3-dihydro-4*H*-benzo[*b*][1,4]oxazin-4yl)acetamido)cyclohexane-1-carboxylate **22** (30 mg, 0.07 mmol) and lithium hydroxide hydrate (21 mg, 0.50 mmol) in methanol (1 mL) and water (0.25 mL) was stirred at 60 °C for 2 h. The reaction mixture was then concentrated under a stream of nitrogen. To the residue was added aqueous HCl (2 M, 5 mL) and DCM (5 mL), the aqueous was extracted with DCM (2 x 5 mL) and the combined organic phases were concentrated under a stream of nitrogen to yield 1-(2-(6-bromo-3-oxo-2,3-dihydro-4*H*-benzo[*b*][1,4]oxazin-4yl)acetamido)cyclohexane-1-carboxylic acid **4** (22 mg, 0.053 mmol, 76%) as a white solid. LCMS (formic) t_R = 0.94 min, *m/z* $[M + H]^+$ = 411, 99% purity; IR ν_{max} (cm^{-1}) 3281, 2935, 2857, 1738, 1656, 1556, 1496, 1445; ^1H NMR (400 MHz, DMSO-d_6) δ 12.21 (br. s, 1H), 8.25 (s, 1H), 7.16 (dd, ^4J = 2.0, J = 8.4 Hz, 1H), 7.09 (d, ^4J = 2.0 Hz, 1H), 6.97 (d, J = 8.4 Hz, 1H), 4.70 (s, 2H), 4.59 (s, 2H), 2.07-1.90 (m, 2H), 1.68-1.44 (m, 7H), 1.30-1.15 (m, 1H); ^{13}C NMR (101 MHz, DMSO-d_6) δ 175.1, 165.7, 163.9, 143.8, 130.4, 125.8, 118.1, 117.9, 113.9, 66.7, 58.2, 42.9, 31.6, 24.9, 20.9; HRMS ($C_{17}H_{20}{}^{79}BrN_2O_5$): $[M + H]^+$ calculated 411.0556, found 411.0551

Ethyl 1-(2-chloroacetamido)cyclohexane-1-carboxylate (23)

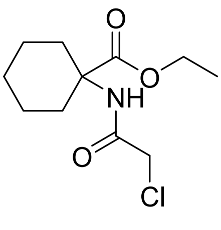

Ethyl 1-aminocyclohexane-1-carboxylate hydrochloride (949 mg, 4.57 mmol) and triethylamine (1.91 mL, 13.7 mmol) in DCM (30 mL) were treated at 0 °C with 2-chloroacetyl chloride (0.473 mL, 5.94 mmol) dropwise and the reaction mixture stirred for 3 h. The reaction mixture was treated with water (50 mL) and DCM (75 mL) and the organic phase isolated by

filtration through a hydrophobic frit. The aqueous phase was extracted with further DCM (75 mL) and the combined organic phases passed through a hydrophobic frit then concentrated under reduced pressure. The residue was purified by Si column chromatography (0-50% EtOAc/cyclohexane) to yield ethyl 1-(2-chloroacetamido)cyclohexane-1-carboxylate **23** as a white solid (765 mg, 3.09 mmol, 68%). LCMS (formic) t_R = 0.89 min, *m/z* $[M + H]^+$ = 248; ^1H NMR (400 MHz, $CDCl_3$) δ 6.68 (br. s, 1H), 4.19 (q, *J* = 7.1 Hz, 2H), 4.04 (s, 2H), 2.13-2.04 (m, 2H), 1.94-1.84 (m, 2H), 1.75-1.61 (m, 3H), 1.50-1.31 (m, 3H), 1.26 (t, *J* = 7.1 Hz, 3H)

1-(2-(3-oxo-6-(trifluoromethyl)-2,3-dihydro-4*H*-benzo[*b*][1,4]oxazin-4-yl)acetamido)cyclohexane-1-carboxylic acid (5)

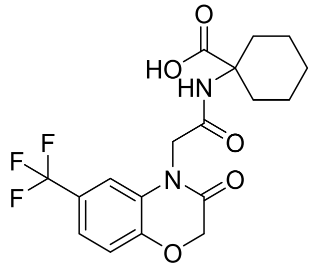

Ethyl 1-(2-chloroacetamido)cyclohexane-1-carboxylate **23** (42 mg, 0.170 mmol), 18-crown-6 (2.6 mg, 9.84 μmol), 6-(trifluoromethyl)-2*H*-benzo[*b*][1,4]oxazin-3(4*H*)-one (37 mg, 0.170 mmol) and K_2CO_3 (35 mg, 0.253 mmol) were stirred in anhydrous acetonitrile (1 mL) at 60 °C for 22 h 50 min. Lithium hydroxide hydrate (28.5 mg, 0.678 mmol), ethanol (0.5 mL) and water (0.5 mL) were added and the reaction stirred at 60 °C for 7.5 h. The reaction mixture was concentrated under a stream of nitrogen. To the residue was added water (2 mL) and 2 M aq. HCl (1 mL) cautiously followed by DCM (5 mL). The layers were separated in a hydrophobic frit and the aqueous layer extracted with an additional portion of DCM (5 mL). The organic layers were combined and solvent removed under a stream of nitrogen. The residue was purified by reverse phase MDAP (formic acid). Fractions containing product were concentrated under a stream of nitrogen to yield 1-(2-(3-oxo-6-(trifluoromethyl)-2,3-dihydro-4*H*-benzo[*b*][1,4]oxazin-4-yl)acetamido)cyclohexane-1-carboxylic acid **5** (36 mg, 0.090 mmol, 53% yield). LCMS (formic) t_R = 0.97 min, *m/z* $[M + H]^+$ = 401, 98% purity; IR ν_{max} (cm^{-1}) 3284, 3070, 2941, 2865, 1741, 1699, 1678, 1651 ,1549, 1519, 1453, 1426; ^1H NMR (400 MHz, DMSO-d_6) δ 12.17 (br. s, 1H), 8.26 (s, 1H), 7.39-7.35 (m, 1H), 7.22-7.17 (m, 2H), 4.80 (s, 2H), 4.66 (s, 2H), 2.02-1.90 (m, 2H), 1.70-1.58 (m, 2H), 1.58-1.39 (m, 5H), 1.30-1.16 (m, 1H); ^{13}C NMR (101 MHz, DMSO-d_6) δ 175.1, 165.7, 163.7, 147.4, 129.2, 123.2 (q, $^2J_{C\text{-}F}$ = 32.3 Hz),

124.0 ($^1J_{C-F}$ app. d, J = 272.2 Hz), 120.7-120.8 (m), 117.1, 112.1-112.3 (m), 66.7, 58.2, 42.8, 31.6, 24.9, 20.8; ^{19}F NMR (376 MHz, DMSO-d_6) δ -60.2 (s, 3F); HRMS ($C_{18}H_{20}F_3N_2O_5$): [M + H]$^+$ calculated 401.1324, found 401.1331

Methyl 4-(2-(6-bromo-3-oxo-2,3-dihydro-4*H*-benzo[*b*][1,4]oxazin-4-yl)acetamido)tetrahydro-2*H*-thiopyran-4-carboxylate (24)

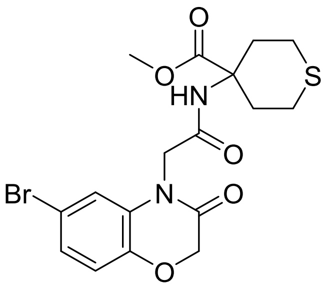

Methyl 4-aminotetrahydro-2*H*-thiopyran-4-carboxylate (371 mg, 2.12 mmol) and triethylamine (0.590 mL, 4.23 mmol) in DCM (10 mL) at 0 °C under a nitrogen atmosphere was treated with 2-chloroacetyl chloride (0.202 mL, 2.54 mmol) dropwise and the reaction stirred for 1 h. The reaction mixture was added treated with water (30 mL) and DCM (25 mL) and the organic phase isolated by filtration through a hydrophobic frit. The aqueous phase was extracted with further DCM (25 mL) and the combined organic phases concentrated under reduced pressure. 6-Bromo-2*H*-benzo[*b*][1,4]oxazin-3(4*H*)-one (483 mg, 2.11 mmol), 18-crown-6 (28.0 mg, 0.106 mmol), K_2CO_3 (441 mg, 3.19 mmol) and MeCN (15 mL) were added and the reaction mixture stirred at 60 °C for 42 h. The reaction mixture was cooled to rt then treated with water (100 mL), brine (10 mL) and DCM (100 mL). The layers were separated and the aqueous phase was extracted with further DCM (100 mL). The combined organic phases were passed through a hydrophobic frit and solvent removed under reduced pressure. The residue was purified by silica chromatography (0-50% EtOAc/cyclohexane) to yield methyl 4-(2-(6-bromo-3-oxo-2,3-dihydro-4*H*-benzo[*b*][1,4]oxazin-4-yl)acetamido)tetrahydro-2*H*-thiopyran-4-carboxylate **24** as a white solid (699 mg, 1.58 mmol, 75%). LCMS (formic) t_R = 0.99 min, *m/z* [M + H]$^+$ = 443; IR ν_{max} (cm^{-1}) 3305, 2947, 1732, 1699, 1666, 1543, 1497, 1430; ^{1}H NMR (400 MHz, DMSO-d_6) δ 8.51 (s, 1H), 7.18 (dd, ^4J = 2.3, J = 8.7 Hz, 1H), 7.08 (d, ^4J = 2.3 Hz, 1H), 6.98 (d, J = 8.7 Hz, 1H), 4.71 (s, 2H), 4.64 (s, 2H), 3.55 (s, 3H), 2.91-2.81 (m, 2H), 2.55-2.47 (m, 2H), 2.30-2.19 (m, 2H), 2.05-1.90 (m, 2H); ^{13}C NMR (101 MHz, DMSO-d_6) δ 173.3, 166.2, 163.9, 143.9, 130.3, 125.9, 118.2, 117.7, 113.8, 66.7, 57.4, 51.9, 42.8, 32.8, 22.4; HRMS ($C_{17}H_{20}{}^{79}BrN_2O_5S$): [M + H]$^+$ calculated 443.0276, found 443.0271

4-(2-(6-Bromo-3-oxo-2,3-dihydro-4*H*-benzo[*b*][1,4]oxazin-4-yl)acetamido)tetrahydro-2*H*-thiopyran-4-carboxylic acid (6)

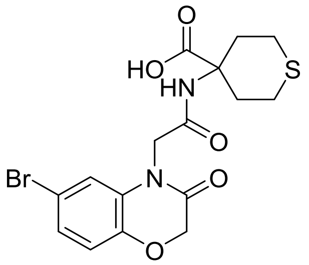

Methyl 4-(2-(6-bromo-3-oxo-2,3-dihydro-4*H*-benzo[*b*][1,4]oxazin-4-yl)acetamido)tetrahydro-2*H*-thiopyran-4-carboxylate **24** (20 mg, 0.045 mmol) in water (0.2 mL) was treated with HCl (4 M in 1,4-dioxane, 1 mL, 4 mmol) and the reaction mixture stirred at 60 °C for 30 h. The solvent was then removed under a stream of nitrogen then the residue was purified by MDAP (formic method) to yield 4-(2-(6-bromo-3-oxo-2,3-dihydro-4*H*-benzo[*b*][1,4]oxazin-4-yl)acetamido)tetrahydro-2*H*-thiopyran-4-carboxylic acid **6** as a white solid (11 mg, 0.026 mmol, 57%). LCMS (formic) t_R = 0.89 min, *m/z* $[M + H]^+$ = 429/431, 100% purity; IR ν_{max} (cm^{-1}) 3307, 2941, 1661, 1548, 1496; ^1H NMR (400 MHz, DMSO-d_6) δ 8.29 (br. s, 1H), 7.16 (dd, ^4J = 2.3, J = 8.4 Hz, 1H), 7.13 (d, ^4J = 2.3 Hz, 1H), 6.96 (d, J = 8.4 Hz, 1H), 4.69 (s, 2H), 4.61 (s, 2H), 2.86-2.75 (m, 2H), 2.57-2.48 (m, 2H), 2.36-2.24 (m, 2H), 1.99-1.87 (m, 2H), *acid proton not observed*; HRMS ($C_{16}H_{21}{}^{79}BrN_3O_5S$): $[M + NH_4]^+$ calculated 446.0385, found 446.0363

Methyl 1-(2-(6-bromo-3-oxo-2,3-dihydro-4*H*-benzo[*b*][1,4]oxazin-4-yl)acetamido)-4,4-difluorocyclohexane-1-carboxylate (25)

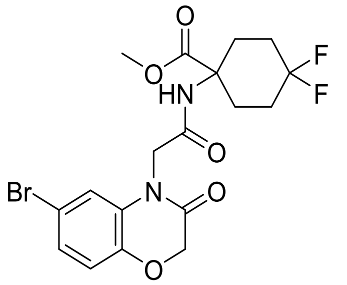

2-(6-bromo-3-oxo-2,3-dihydro-4*H*-benzo[*b*][1,4]oxazin-4-yl)acetic acid **21** (50 mg, 0.18 mmol) was treated with methyl 1-amino-4,4-difluorocyclohexane-1-carboxylate (40 mg, 0.21 mmol), HATU (80 mg, 0.21 mmol) and DMF (1 mL) and the reaction mixture stirred at rt for 1 h 15 min. The reaction mixture was treated with DIPEA (45 mg, 0.35 mmol) and stirred at rt

for 3 h 15 min. The reaction mixture was treated with methyl 1-amino-4,4-difluorocyclohexane-1-carboxylate (40 mg, 0.21 mmol), HATU (80 mg, 0.21 mmol) and DIPEA (45 mg, 0.35 mmol) and stirred for 1 h. The reaction mixture was treated with DCM (5 mL) and water (5 mL) and the organic phase isolated by filtration through a hydrophobic frit. The aqueous phase was extracted with additional DCM (2 × 5 mL) and the combined organic phases concentrated under a stream of nitrogen. The residue was purified by MDAP (formic method) to yield methyl 1-(2-(6-bromo-3-oxo-2,3-dihydro-4*H*-benzo[*b*][1,4]oxazin-4-yl)acetamido)-4,4-difluorocyclohexane-1-carboxylate **25** as a white solid (41 mg, 0.089 mmol, 51%). LCMS (formic) t_R = 1.04 min, *m/z* $[M + H]^+$ = 461; ^{1}H NMR (400 MHz, DMSO-d_6) δ 8.65 (s, 1H), 7.18 (dd, ^4J = 2.5, J = 8.7 Hz, 1H), 7.09 (d, ^4J = 2.5 Hz, 1H), 6.98 (d, J = 8.7 Hz, 1H), 4.71 (s, 2H), 4.63 (s, 2H), 3.56 (s, 3H), 2.16-1.87 (m, 8H).

1-(2-(6-bromo-3-oxo-2,3-dihydro-4*H*-benzo[*b*][1,4]oxazin-4-yl)acetamido)-4,4-difluorocyclohexane-1-carboxylic acid (7)

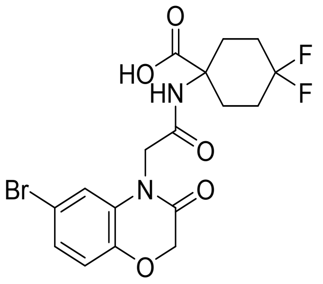

Methyl 1-(2-(6-bromo-3-oxo-2,3-dihydro-4*H*-benzo[*b*][1,4]oxazin-4-yl)acetamido)-4,4-difluorocyclohexane-1-carboxylate **25** (41 mg, 0.089 mmol) and lithium hydroxide hydrate (43 mg, 1.025 mmol) in methanol (1.5 mL) and water (0.5 mL) were stirred at 60 °C for 1 h. The reaction mixture was concentrated under a stream of nitrogen. To the residue was added 2M aq. HCl (5 mL) followed by DCM (5 mL) and the layers separated in a hydrophobic frit. The aqueous layer was extracted with an additional portion of DCM (5 x 5 mL) and the combined organic layers concentrated under a stream of nitrogen yielding 1-(2-(6-bromo-3-oxo-2,3-dihydro-4*H*-benzo[*b*][1,4]oxazin-4-yl)acetamido)-4,4-difluorocyclohexane-1-carboxylic acid **7** (28 mg, 0.063 mmol, 70 % yield) as a white solid. LCMS (formic) t_R = 0.94 min, *m/z* $[M + H]^+$ = 447, 100% purity; IR ν_{max} (cm^{-1}) 3286, 2937, 1667, 1545, 1496, 1439; ^{1}H NMR (400 MHz, DMSO-d_6) δ 12.59 (br. s, 1H), 8.48 (s, 1H), 7.16 (dd, ^4J = 2.0, J = 8.4 Hz, 1H), 7.13 (d, ^4J = 2.0 Hz, 1H), 6.97 (d, J = 8.4 Hz, 1H), 4.70 (s, 2H), 4.62 (s, 2H), 2.22-2.12 (m, 2H), 2.11-1.85 (m, 6H); ^{13}C NMR (DMSO-d_6, 151 MHz) δ 174.1, 166.6, 164.0, 143.9, 130.5, 125.9,

123.3 (br t, ${}^{1}J_{C\text{-}F}$ = 239.9 Hz), 118.2, 117.9, 113.9, 66.8, 56.7, 43.1, 28.9 (t, ${}^{2}J_{C\text{-}F}$ = 24.1 Hz), 28.4-28.5 (app. d); ^{19}F NMR (376 MHz, DMSO-d_6) δ -90.8 (d, ${}^{2}J_{F\text{-}F}$ = 235.7 Hz, 1F), -100.1 (d, ${}^{2}J_{F\text{-}F}$ = 228.9 Hz, 1F); HRMS ($C_{17}H_{18}{}^{79}BrF_2N_2O_5$): $[M + H]^+$ calculated 447.0367, found 447.0361.

Methyl 1-(2-chloro-*N*-methylacetamido)cyclohexane-1-carboxylate (26)

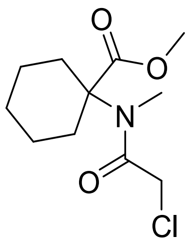

To methyl 1-(methylamino)cyclohexane-1-carboxylate (1 g, 6 mmol) and Et_3N (1.87 mL, 13.4 mmol) in DCM (30 mL) at 0 °C under nitrogen was added 2-chloroacetyl chloride (0.6 mL, 8 mmol) and the reaction mixture was stirred for 1 h at 0 °C under nitrogen. To the reaction mixture was added water (10 mL) followed by DCM (30 mL). The reaction mixture was then passed through a hydrophobic frit the organic phase was collected and the solvent was removed under a stream of nitrogen. The residue was purified by normal phase silica chromatography (0-80% EtOAc/cyclohexane) to yield methyl 1-(2-chloro-*N*-methylacetamido)cyclohexane-1-carboxylate **26** (960 mg, 3.88 mmol, 66%) as a pale peach solid. LCMS (formic) t_R = 0.89 min, *m/z* $[M + H]^+$ = 248; ^{1}H NMR (400 MHz, DMSO-d_6) δ 4.37 (s, 2H), 3.54 (s, 3H), 2.99 (s, 3H), 1.99-1.91 (m, 2H), 1.78-1.67 (m, 2H), 1.62-1.45 (m, 5H), 1.39-1.22 (m, 1H)

1-(2-(6-bromo-3-oxo-2,3-dihydro-4*H*-benzo[*b*][1,4]oxazin-4-yl)-*N*-methylacetamido)cyclohexane-1-carboxylic acid (8)

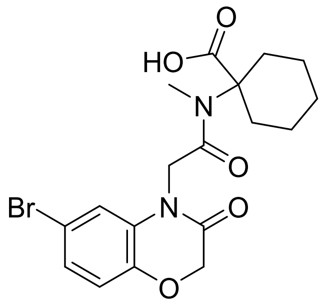

6-bromo-2*H*-benzo[*b*][1,4]oxazin-3(4*H*)-one (42 mg, 0.18 mmol), 18-crown-6 (2 mg, 8 μmol), methyl 1-(2-chloro-*N*-methylacetamido)cyclohexane-1-carboxylate **26** (45 mg, 0.18 mmol) and K_2CO_3 (38 mg, 0.28 mmol) were stirred in MeCN (1 mL) at 60 °C for 16 h. To the reaction mixture was added water (5 mL) and DCM (5 mL) and the phases separated through a

hydrophobic frit. The aqueous phase was extracted with additional portions of DCM (2 × 5 mL) and the combined organic phases concentrated under a stream of nitrogen. The residue was purified by reverse phase MDAP (formic method). To the isolated ester was added LiOH (21 mg, 0.88 mmol) dissolved in water (0.25 mL) and methanol (0.75 mL) and the reaction stirred at 60 °C for 22 h. DCM (5 mL) and 2M aqueous HCl (5 mL) were added then the reaction mixture concentrated under a stream of nitrogen. The residue was purified by reverse phase MDAP (formic method) to yield 1-(2-(6-bromo-3-oxo-2,3-dihydro-4*H*-benzo[*b*][1,4]oxazin-4-yl)-*N*-methylacetamido)cyclohexane-1-carboxylic acid **8** (5.3 mg, 0.012 mmol, 7%) as a white solid. LCMS (formic) t_R = 1.01 min, *m/z* $[M - H]^-$ = 423, 100% purity; ^1H NMR (400 MHz, DMSO-d_6) δ 7.14 (dd, *J* = 8.5, 2.2 Hz, 1H), 7.00 (d, ^4J = 2.2 Hz, 1H), 6.96 (d, *J* = 8.5 Hz, 1H), 4.81 (s, 2H), 4.68 (s, 2H), 3.09 (s, 3H), 1.94-1.82 (m, 4H), 1.66-1.53 (m, 2H), 1.53-1.33 (m, 4H) *COOH proton not observed.*

Ethyl 1-(2-(6-bromo-3-oxo-2,3-dihydro-4*H*-benzo[*b*][1,4]oxazin-4-yl)acetamido)cyclohexane-1-carboxylate (27)

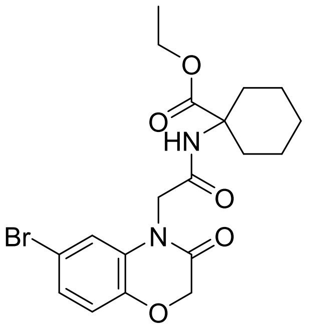

Ethyl 1-(2-chloroacetamido)cyclohexane-1-carboxylate **23** (200 mg, 0.807 mmol) was treated with 18-crown-6 (10 mg, 0.040 mmol), K_2CO_3 (168 mg, 1.22 mmol) and 6-bromo-2*H*-benzo[*b*][1,4]oxazin-3(4*H*)-one (184 mg, 0.807 mmol) and MeCN (8.07 mL) then stirred at 60 °C for 1 h. The solvent was removed under a stream of nitrogen. The residue was treated with aqueous 2 M HCl (5 mL), water (3 mL) and DCM (5 mL) and the organic phase isolated by filtration through a hydrophobic frit. The aqueous phase was extracted with further DCM (10 mL) and concentrated under a stream of nitrogen to yield ethyl 1-(2-(6-bromo-3-oxo-2,3-dihydro-4*H*-benzo[*b*][1,4]oxazin-4-yl)acetamido)cyclohexane-1-carboxylate **27** as a pale brown solid (330 mg, 0.751 mmol, 93%). LCMS (HpH) t_R = 1.17 min, *m/z* $[M + H]^+$ = 439, 94% purity; ^1H NMR (400 MHz, DMSO-d_6) δ 8.39 (s, 1H), 7.17 (dd, ^2J = 2.5, *J* = 8.4 Hz, 1H), 7.08 (d, ^4J = 2.5 Hz, 1H), 6.97 (d, *J* = 8.4 Hz, 1H), 4.70 (s, 2H), 4.60 (s, 2H), 3.99 (q, *J* = 7.0

Hz, 2H), 1.99-1.89 (m, 2H), 1.72-1.61 (m, 2H), 1.60-1.44 (m, 5H), 1.33-1.18 (m, 1H), 1.06 (t, J = 7.0 Hz, 3H).

Ethyl 1-(2-(3-oxo-6-phenyl-2,3-dihydro-4*H*-benzo[*b*][1,4]oxazin-4-yl)acetamido)cyclohexane-1-carboxylate (28)

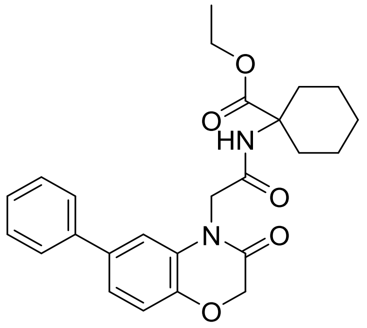

Ethyl 1-(2-(6-bromo-3-oxo-2,3-dihydro-4*H*-benzo[*b*][1,4]oxazin-4-yl)acetamido)cyclohexane-1-carboxylate **27** (40 mg, 0.091 mmol), 4,4,5,5-tetramethyl-2-phenyl-1,3,2-dioxaborolane (25 mg, 0.12 mmol), K_2CO_3 (50.3 mg, 0.364 mmol), bis(triphenylphosphine)palladium(II) chloride (6 mg, 8 μmol), water (0.1 mL) and DMF (2 mL) were added to a vial. The vial was evacuated and purged three times with nitrogen. The reaction mixture was heated to 100 °C in a microwave reactor for 2 h. The reaction mixture was treated with saturated aqueous $NaHCO_3$ (5 mL) and DCM (15 mL) and the organic phase isolated by filtration through a hydrophobic frit. The solvent was concentrated under a stream of nitrogen. The residue was purified by MDAP (HpH method) to yield ethyl 1-(2-(3-oxo-6-phenyl-2,3-dihydro-4*H*-benzo[*b*][1,4]oxazin-4-yl)acetamido)cyclohexane-1-carboxylate **28** (17 mg, 0.039 mmol, 43%) as a white solid. LCMS (formic) t_R = 1.21 min, m/z $[M + H]^+$ = 437, 100% purity; ^1H NMR (400 MHz, DMSO-d_6) δ 8.42 (s, 1H), 7.68-7.62 (m, 2H), 7.48-7.40 (m, 2H), 7.37-7.32 (m, 1H), 7.30 (dd, ^4J = 2.0, J = 7.9 Hz, 1H), 7.20 (d, ^4J = 2.0 Hz, 1H), 7.09 (d, J = 8.4 Hz, 1H), 4.73 (s, 2H), 4.70 (s, 2H), 3.80 (q, J = 7.1 Hz, 2H), 1.98-1.88 (m, 2H), 1.71-1.58 (m, 2H), 1.51-1.42 (m, 4H), 1.29-1.13 (m, 2H), 0.88 (t, J = 7.1 Hz, 3H).

1-(2-(3-oxo-6-phenyl-2,3-dihydro-4*H*-benzo[*b*][1,4]oxazin-4-yl)acetamido)cyclohexane-1-carboxylic acid (9)

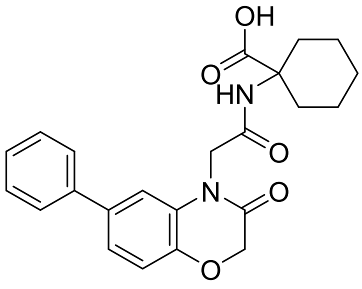

A solution of ethyl 1-(2-(3-oxo-6-phenyl-2,3-dihydro-4*H*-benzo[*b*][1,4]oxazin-4-yl)acetamido)cyclohexane-1-carboxylate **28** (17 mg, 0.039 mmol), LiOH hydrate (10 mg, 0.418 mmol), ethanol (0.5 mL) and water (0.5 mL) was stirred for 72 h at 60 °C. To the reaction mixture was added aq. HCL (2 mL), water (3 mL) and DCM (15 mL). The solution was passed through a phase separator and the solvent was removed under a stream of N_2 to yield 1-(2-(3-oxo-6-phenyl-2,3-dihydro-4H-benzo[b][1,4]oxazin-4-yl)acetamido)cyclohexane-1-carboxylic acid **9** as a white solid (11 mg, 0.026 mmol, 66%). LCMS (formic) t_R = 1.05 min, *m/z* $[M + H]^+$ = 409, 100% purity; IR ν_{max} (cm^{-1}) 3676, 3286, 2938, 2859, 1698, 1678, 1658, 1548, 1487, 1433; ^1H NMR (400 MHz, DMSO-d_6) δ 12.19 (br. s, 1H), 8.26 (s, 1H), 7.69-7.60 (m, 2H), 7.46-7.40 (m, 2H), 7.36-7.31 (m, 1H), 7.29 (dd, ^4J = 2.0, J = 8.3 Hz, 1H), 7.19 (d, ^4J = 2.0 Hz, 1H), 7.08 (d, J = 8.3 Hz, 1H), 4.72 (s, 2H), 4.68 (s, 2H), 2.03-1.93 (m, 2H), 1.70-1.56 (m, 2H), 1.48-1.37 (m, 4H), 1.27-1.13 (m, 2H); ^{13}C NMR (101 MHz, DMSO-d_6) δ 175.2, 166.0, 164.0, 144.1, 139.5, 134.9, 129.1, 128.7, 127.1, 126.5, 121.7, 116.7, 113.6, 66.9, 58.2, 42.9, 31.6, 24.8, 20.9; HRMS ($C_{23}H_{25}N_2O_5$): $[M + H]^+$ calculated 409.1763, found 409.1769

Methyl 1-(2-chloroacetamido)cyclohexane-1-carboxylate (29)

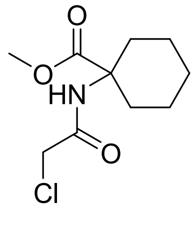

To methyl 1-aminocyclohexane-1-carboxylate (5 g, 31.8 mmol) and triethylamine (8.87 mL, 63.6 mmol) in anhydrous DCM (170 mL) at 0 °C under nitrogen was added 2-chloroacetyl chloride (3.04 mL, 38.2 mmol) dropwise and the reaction stirred for 1 h under N_2. To the reaction mixture was added water (200 mL) and DCM (20 mL). The phases were separated, the organic layer collected and the solvent was removed under a stream of N_2. The residue was

then loaded in DCM and absorbed onto a 120g Si column. The residue was eluted with ethyl acetate/cyclohexane (0-50%). The fractions containing product were collected and the solvent was removed under a stream of N_2 to yield methyl 1-(2-chloroacetamido)cyclohexane-1-carboxylate **29** (4.98 g, 21.31 mmol, 67 % yield). LCMS (formic): rt: 0.79 min, *m/z* $[M + H]^+$ = 234; ^1H NMR (400 MHz, DMSO-d_6) δ = 8.30 (s, 1H), 4.08 (s, 2H), 3.57 (s, 3H), 1.98 - 1.88 (m, 2H), 1.77 - 1.61 (m, 2H), 1.57 - 1.41 (m, 5H), 1.35 - 1.15 (m, 1H).

Methyl 1-(2-(5-bromo-2-oxobenzo[*d*]oxazol-3(2*H*)-yl)acetamido)cyclohexane-1-carboxylate (30)

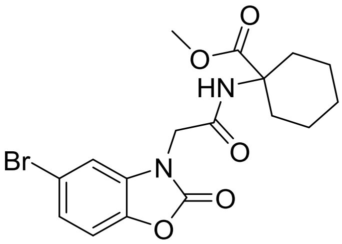

5-bromobenzo[*d*]oxazol-2(3*H*)-one (0.900 g, 4.21 mmol), methyl 1-(2-chloroacetamido)cyclohexane-1-carboxylate **29** (1 g, 4.28 mmol), 18- crown-6 (0.100 g, 0.378 mmol) and K_2CO_3 (0.9 g, 6.51 mmol) were stirred in anhydrous MeCN (20 mL) at 60 °C for 16 h. The reaction mixture was then cooled to room temp then filtered and the solid was washed with water (35 mL). The solid was dried in a vacuum oven to yield methyl 1-(2-(5-bromo-2-oxobenzo[*d*]oxazol-3(2*H*)-yl)acetamido)cyclohexane-1-carboxylate **30** (1 g, 2.432 mmol, 56.8 % yield) as a pale brown solid. LCMS (formic) rt:1.09 min, *m/z* $[M + H]^+$ = 411, 100% purity; ^1H NMR (400 MHz, DMSO-d_6) δ = 8.57 (s, 1H), 7.39 - 7.37 (m, 1H), 7.36 - 7.30 (m, 2H), 4.57 (s, 2H), 3.54 (s, 3H), 2.01 - 1.89 (m, 2H), 1.76 - 1.62 (m, 2H), 1.61 - 1.47 (m, 5H), 1.35 - 1.18 (m, 1H).

1-(2-(5-bromo-2-oxobenzo[*d*]oxazol-3(2*H*)-yl)acetamido)cyclohexane-1-carboxylic acid (10)

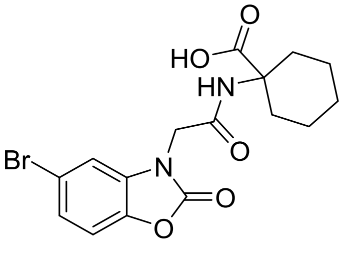

A solution of methyl 1-(2-(5-bromo-2-oxobenzo[*d*]oxazol-3(2*H*)-yl)acetamido)cyclohexane-1-carboxylate **30** (70 mg, 0.170 mmol), HCl (4 M in 1,4-dioxane) (851 µl, 3.40 mmol) and

water (340 µl) was heated to 60 °C and stirred for 18 h. The reaction was cooled to rt and evaporated to dryness. The samples were dissolved in water/DMSO and purified by MDAP (formic method). The solvent was dried under a stream of nitrogen to give 1-(2-(5-bromo-2-oxobenzo[*d*]oxazol-3(2*H*)-yl)acetamido)cyclohexane-1-carboxylic acid **10** (31 mg, 0.078 mmol, 46 % yield) as a white solid. LCMS (formic): t_R = 0.95 min, *m/z* $[M + H]^+$ = 397, (99% purity); ^1H NMR (400 MHz, DMSO-d_6) δ = 12.24 (s, 1H), 8.31 (s, 1H), 7.40 (d, *J* = 1.0 Hz, 1H), 7.34 - 7.29 (m, 2H), 4.55 (s, 2H), 2.02 - 1.93 (m, 2H), 1.71 - 1.45 (m, 7H), 1.32 - 1.17 (m, 1H).

1-(2-(2-Oxo-5-phenylbenzo[*d*]oxazol-3(2*H*)-yl)acetamido)cyclohexane-1-carboxylic acid (11)

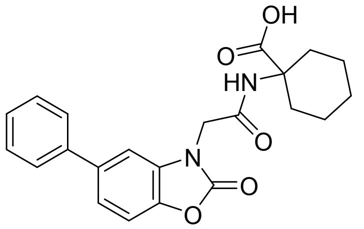

A microwave vial was charged with methyl 1-(2-(5-bromo-2-oxobenzo[*d*]oxazol-3(2*H*)-yl)acetamido)cyclohexane-1-carboxylate **30** (50 mg, 0.12 mmol), 4,4,5,5-tetramethyl-2-phenyl-1,3,2-dioxaborolane (50 mg, 0.25 mmol), K_2CO_3 (50 mg, 0.36 mmol), Pd(amphos)Cl_2 (8 mg, 0.01 mmol), 1,4-dioxane (1 mL) and water (0.2 mL). The vial was evacuated and purged with nitrogen three times then heated in a microwave reactor at 80 °C for 1 h. To the reaction mixture was added water (5 mL) and DCM (5 mL) and the phases separated using a hydrophobic frit. The aqueous phase was extracted with additional portions of DCM (2 × 5 mL) and the combined organic phases concentrated under a stream of nitrogen. The residue was purified by MDAP (formic method) to yield the ester intermediate. To the ester intermediate was added 4 M HCl in 1,4-dioxane (1 mL, 4 mmol) and water (0.2 mL) and the reaction stirred at 60 °C for 66 h. The reaction mixture was concentrated under a stream of nitrogen then purified by MDAP (formic method) to yield 1-(2-(2-oxo-5-phenylbenzo[*d*]oxazol-3(2*H*)-yl)acetamido)cyclohexane-1-carboxylic acid **11** as a white solid (8 mg, 0.02 mmol, 17%). LCMS (formic) t_R = 1.05 min, *m/z* $[M-H]^-$ = 393, 99% purity; ^1H NMR (400 MHz, DMSO-d_6) δ 12.24 (br. s, 1H), 8.34 (s, 1H), 7.67-7.62 (m, 2H), 7.50-7.44 (m, 2H), 7.43-7.40 (m, 3H), 7.39-7.34 (m, 1H), 4.60 (s, 2H), 2.03-1.94 (m, 2H), 1.73-1.60 (m, 2H), 1.59-1.45 (m, 5H), 1.31-1.16 (m, 1H).

1-(2-(6-bromo-2,3-dioxoindolin-1-yl)acetamido)cyclohexane-1-carboxylate (31)

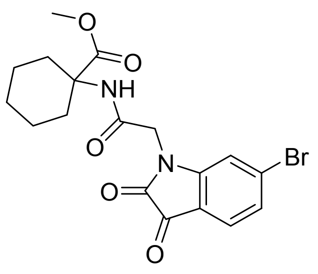

6-bromoindoline-2,3-dione (513 mg, 2.27 mmol), 18-crown-6 (20 mg, 0.076 mmol), methyl 1-(2-chloroacetamido)cyclohexane-1-carboxylate **29** (530 mg, 2.27 mmol) and K_2CO_3 (470 mg, 3.40 mmol) were stirred in anhydrous MeCN (20 mL) at 60 °C for 22 h. To the reaction mixture was added additional K_2CO_3 (200 mg, 1.45 mmol) and 18-crown-6 (20 mg, 0.076 mmol) and the reaction stirred at 60 °C for 4 h. The solvent was removed under reduced pressure. The residue was purified by normal phase silica chromatography (0-50% EtOAc/cyclohexane) to yield 1-(2-(6-bromo-2,3-dioxoindolin-1-yl)acetamido)cyclohexane-1-carboxylate as a yellow solid **31** (292 mg, 0.690 mmol, 30%). LCMS (formic) t_R = 1.01 min, *m/z* $[M + H]^+$ = 423; ^1H NMR (400 MHz, DMSO-d_6) δ 8.33 (s, 1H), 7.52 (d, *J* = 8.0 Hz, 1H), 7.37 (dd, ^4J = 1.6, *J* = 8.0 Hz, 1H), 7.26 (d, ^4J = 1.6 Hz, 1H), 4.42 (s, 2H), 3.54 (s, 3H), 2.00-1.90 (m, 2H), 1.73-1.62 (m, 2H), 1.59-1.39 (m, 5H), 1.32-1.18 (m, 1H).

Methyl 1-(2-(6-bromo-3,3-dichloro-2-oxoindolin-1-yl)acetamido)cyclohexane-1-carboxylate (32)

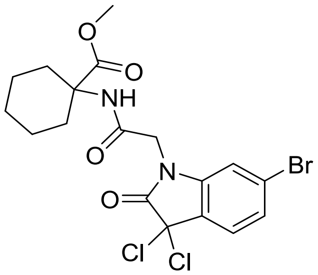

To a solution of methyl 1-(2-(6-bromo-2,3-dioxoindolin-1-yl)acetamido)cyclohexane-1-carboxylate **31** (241 mg, 0.569 mmol) in toluene (15 mL) was added PCl_5 (261 mg, 1.25 mmol) and the reaction stirred at 60 °C for 16 h. The reaction was cooled to rt then PCl_5 (46 mg, 0.22 mmol) added. The reaction was stirred at 60 °C for 1 h. The reaction was cooled to rt then added slowly to sodium acetate (700 mg) dissolved in water (50 mL) and stirred for 10 min. To this was added DCM (30 mL) and the phases separated. The aqueous phase was extracted with an additional portion of DCM (30 mL). The combined organic phases were passed through a hydrophobic frit and the solvent removed under reduced pressure. The residue was purified

by normal phase silica chromatography (0-50% TBME/cyclohexane) to yield methyl 1-(2-(6-bromo-3,3-dichloro-2-oxoindolin-1-yl)acetamido)cyclohexane-1-carboxylate **32** as a white solid (140 mg, 0.293 mmol, 51%). LCMS (formic) t_R = 1.22 min, *m/z* $[M + H]^+$ = 477; ^1H NMR (400 MHz, DMSO-d_6) δ 8.51 (s, 1H), 7.69 (d, J = 8.2 Hz, 1H), 7.44 (dd, ^4J = 1.6, J = 8.2, 1H), 7.24 (d, J = 1.6 Hz, 1H), 4.51 (s, 2H), 3.53 (s, 3H), 1.99-1.89 (m, 2H), 1.77-1.63 (m, 2H), 1.62-1.47 (m, 5H), 1.32-1.21 (m, 1H).

Methyl 1-(2-(6-bromo-2-oxoindolin-1- yl)acetamido)cyclohexane-1-carboxylate (33)

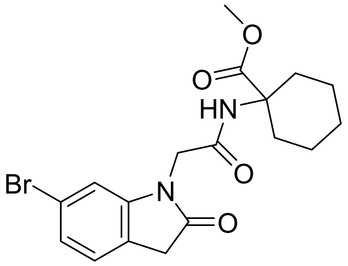

To a solution of methyl 1-(2-(6-bromo-3,3-dichloro-2-oxoindolin-1-yl)acetamido)cyclohexane-1-carboxylate **32** (82 mg, 0.171 mmol) in acetic acid (1 mL) was added zinc (123 mg, 1.886 mmol) and the reaction stirred under nitrogen for 30 min. The reaction was filtered and the filter washed with EtOAc (5 mL). The filtrate was concentrated under a stream of nitrogen. To the reaction mixture was added sat. aq. $NaHCO_3$ (5 mL) and DCM (5 mL). The layers were separated in a hydrophobic frit and the aqueous layer extracted with additional portions of DCM (2 × 5 mL). Solvent was removed under a stream of nitrogen to yield methyl 1-(2-(6-bromo-2-oxoindolin-1-yl)acetamido)cyclohexane-1-carboxylate **33** (59 mg, 0.144 mmol, 84 % yield) as a colourless gum. LCMS (formic) t_R =1.01 min, *m/z* $[M + H]^+$ = 409, 92% purity; ^1H NMR (400 MHz, DMSO-d_6) δ = 8.39 (s, 1H), 7.21 (d, J = 7.8 Hz, 1H), 7.18 (dd, J = 1.6, 7.8 Hz, 1H), 6.98 (d, J = 1.6 Hz, 1H), 4.37 (s, 2H), 3.57 (s, 2H), 3.53 (s, 3H), 2.00 - 1.88 (m, 2H), 1.73 - 1.44 (m, 7H), 1.31 - 1.20 (m, 1H).

1-(2-(2-Oxo-6-phenylindolin-1-yl)acetamido)cyclohexane-1-carboxylic acid (12)

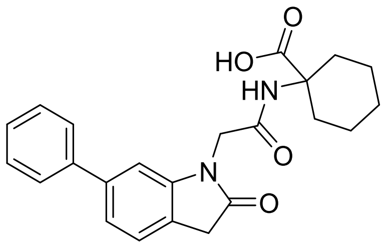

A microwave vial was charged with methyl 1-(2-(6-bromo-2-oxoindolin-1-yl)acetamido)cyclohexane-1-carboxylate **33** (45 mg, 0.11 mmol), 4,4,5,5-tetramethyl-2-phenyl-1,3,2-dioxaborolane (45 mg, 0.22 mmol), K_2CO_3 (45 mg, 0.33 mmol), Pd(amphos)Cl_2 (7 mg, 0.01 mmol), 1,4-dioxane (1 mL) and water (0.2 mL). The vial was evacuated and purged with nitrogen three times then heated in a microwave reactor at 80 °C for 1.5 h. To the reaction mixture was added water (5 mL) and DCM (5 mL) and the phases separated using a hydrophobic frit. The aqueous phase was extracted with additional portions of DCM (2 × 5 mL) and the combined organic phases concentrated under reduced pressure. To the residue was added 4 M HCl in 1,4-dioxane (0.8 mL, 3 mmol) and the reaction mixture stirred at 60 °C for 2.5 h. To the reaction mixture was added water (0.2 mL) and the reaction mixture stirred at 60 °C for 20.5 h. The reaction mixture was concentrated under a stream of nitrogen. The residue was purified MDAP (formic method) to yield 1-(2-(2-Oxo-6-phenylindolin-1-yl)acetamido)cyclohexane-1-carboxylic acid **12** as a white solid (12 mg, 0.031 mmol, 28%). LCMS (formic) t_R = 1.01 min, *m/z* $[M + H]^+$ = 393, 100% purity; IR ν_{max} (cm^{-1}) 3707, 3297, 2983, 2935, 2849, 1729, 1656, 1621, 1545, 1489 1432; ^1H NMR (400 MHz, DMSO-d_6) δ 12.21 (br. s, 1H), 8.23 (s, 1H), 7.67-7.61 (m, 2H), 7.48-7.42 (m, 2H), 7.39-7.31 (m, 2H), 7.28 (dd, ^4J = 1.4, J = 7.6 Hz, 1H), 7.08 (d, ^4J = 1.4 Hz, 1H), 4.42 (s, 2H), 3.63 (s, 2H), 2.04-1.95 (m, 2H), 1.71-1.57 (m, 2H), 1.56-1.42 (m, 5H), 1.28-1.17 (m, 1H); ^{13}C NMR (101 MHz, DMSO-d_6) δ 174.5, 174.4, 166.0, 145.0, 140.3, 139.8, 128.8, 127.4, 126.6, 124.4, 123.6, 120.2, 107.1, 58.3, 41.9, 34.8, 31.7, 24.9, 21.0; HRMS ($C_{23}H_{25}N_2O_4$): $[M + H]^+$ calculated 393.1814, found 393.1819.

Methyl 1-(2-(5-chloro-2-oxobenzo[*d*]thiazol-3(2*H*)-yl)acetamido)cyclohexane-1-carboxylate (34)

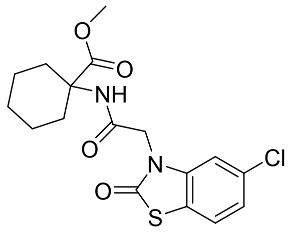

5-chlorobenzo[*d*]thiazol-2(3*H*)-one (560 mg, 3.02 mmol), 18-crown-6 (95 mg, 0.36 mmol), methyl 1-(2-chloroacetamido)cyclohexane-1-carboxylate (700 mg, 3.00 mmol) and K_2CO_3 (620 mg, 4.49 mmol) were stirred in anhydrous MeCN (8 mL) at 60 °C for 16 h. To the reaction was added methyl 1-(2-chloroacetamido)cyclohexane-1-carboxylate (140 mg, 0.599 mmol). The reaction mixture was stirred at 60 °C for 5 h. The solvent was then removed under a stream of nitrogen. The residue was purified by C18 column chromatography (0-100% MeCN/water, formic acid modifier) to yield methyl 1-(2-(5-chloro-2-oxobenzo[*d*]thiazol-3(2*H*)-yl)acetamido)cyclohexane-1-carboxylate **34** as a pale brown solid (924 mg, 2.41 mmol, 81% yield). LCMS (formic) t_R = 1.09 min, *m/z* $[M + H]^+$ = 383; ^1H NMR (400 MHz, DMSO-d_6) δ 8.53 (s, 1H), 7.70 (d, *J* = 8.4 Hz, 1H), 7.27 (dd, ^4J = 2.0, *J* = 8.4 Hz, 1H), 7.21 (d, ^4J = 2.0 Hz, 1H), 4.68 (s, 2H), 3.51 (s, 3H), 2.01-1.90 (m, 2H), 1.75-1.61 (m, 2H), 1.60-1.50 (m, 5H), 1.32-1.19 (m, 1H).

1-(2-(2-Oxo-5-phenylbenzo[*d*]thiazol-3(2*H*)-yl)acetamido)cyclohexane-1-carboxylic acid (13)

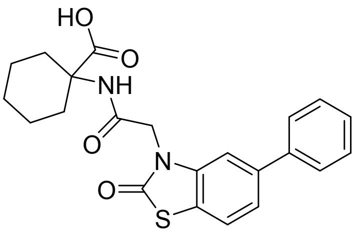

A microwave vial was charged with methyl 1-(2-(5-chloro-2-oxobenzo[*d*]thiazol-3(2*H*)-yl)acetamido)cyclohexane-1-carboxylate **34** (50 mg, 0.13 mmol), 4,4,5,5-tetramethyl-2-phenyl-1,3,2-dioxaborolane (45 mg, 0.22 mmol), K_2CO_3 (45 mg, 0.33 mmol), Pd(amphos)Cl_2 (7 mg, 0.01 mmol), 1,4-dioxane (1 mL) and water (0.2 mL). The vial was evacuated and purged with nitrogen three times then heated in a microwave reactor at 80 °C for 6 h. The reaction mixture was cooled then additional portions of Pd(amphos)Cl_2 (7 mg, 0.01 mmol) and 4,4,5,5-tetramethyl-2-phenyl-1,3,2-dioxaborolane (45 mg, 0.22 mmol) were added. The vial was

evacuated and purged with nitrogen three times then heated in a microwave reactor at 80 °C for 3 h. The reaction mixture was cooled then DCM (5 mL) and water (5 mL) added. The phases were separated using a hydrophobic frit. The aqueous phase was extracted with additional portions of DCM (2 × 5 mL) and the combined organic phases concentrated under a stream of nitrogen. The residue was purified by MDAP (formic method) to yield the ester intermediate. To the ester intermediate was added 4 M HCl in 1,4-dioxane (1 mL, 4 mmol) and water (0.2 mL) and the reaction stirred at 60 °C for 18 h. The reaction was concentrated under a stream of nitrogen then the residue purified by reverse phase MDAP (formic method) to yield 1-(2-(2-oxo-5-phenylbenzo[*d*]thiazol-3(2*H*)-yl)acetamido)cyclohexane-1-carboxylic acid **13** as a white solid (8 mg, 0.02 mmol, 15%). LCMS (formic) t_R = 1.12 min, *m/z* $[M + H]^+$ = 411, 98% purity; IR ν_{max} (cm^{-1}) 3698, 3300, 2947, 1661, 1603, 1550, 1475, 1438; ^1H NMR (400 MHz, DMSO-d_6) δ 12.27 (s, 1H), 8.38 (s, 1H), 7.74 (d, *J* = 8.3 Hz, 1H), 7.72-7.68 (m, 2H), 7.53-7.46 (m, 3H), 7.43-7.36 (m, 2H), 4.74 (s, 2H), 2.05-1.95 (m, 2H), 1.72-1.59 (m, 2H), 1.56-1.45 (m, 5H), 1.31-1.15 (m, 1H); ^{13}C NMR (101 MHz, DMSO-d_6) δ 175.1, 169.2, 165.3, 139.6, 138.9, 137.8, 128.9, 127.6, 126.8, 123.1, 121.7, 120.2, 109.7, 58.3, 44.3, 31.6, 24.9, 20.9; HRMS ($C_{22}H_{23}N_2O_4S$): $[M + H]^+$ calculated 411.1379, found 411.1382.

Methyl 1-(2-(5-(cyclohex-1-en-1-yl)-2-oxobenzo[*d*]thiazol-3(2*H*)-yl)acetamido)cyclohexane-1-carboxylate (36)

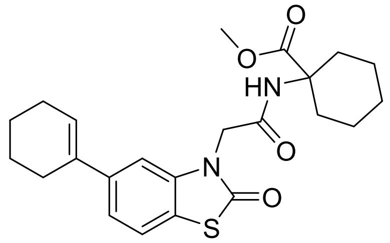

A microwave vial was charged with methyl 1-(2-(5-chloro-2-oxobenzo[*d*]thiazol-3(2*H*)-yl)acetamido)cyclohexane-1-carboxylate **34** (111 mg, 0.290 mmol), cyclohex-1-en-1-ylboronic acid (128 mg, 1.016 mmol), potassium carbonate (141 mg, 1.020 mmol), Pd(amphos)Cl_2 (36 mg, 0.051 mmol), 1,4-dioxane (2.5 mL) and water (0.50 mL). The vial was sparged with nitrogen for 5 minutes, then heated in a microwave reactor at 80 °C for 2 h. Further Pd(amphos)Cl_2 (36 mg, 0.051 mmol) was added, the reaction was sparged with nitrogen for 5 minutes, then heated in a microwave reactor at 80 °C for 2 h. The reaction was filtered through celite, eluting with methanol (5 column volumes), and the combined organics were reduced in vacuo. The crude product was loaded onto celite and purified by normal phase

chromatography (0-100% EtOAc in cyclohexane), using a 12 g silica cartridge. Solvent was evaporated and the residue was loaded onto celite and purified by normal phase chromatography (0-40% EtOAc in cyclohexane), using a 12 g silica cartridge. The desired fractions were combined and reduced in vacuo to give methyl 1-(2-(5-(cyclohex-1-en-1-yl)-2-oxobenzo[*d*]thiazol-3(2*H*)-yl)acetamido)cyclohexane-1-carboxylate **36** (64 mg, 0.119 mmol, 41% yield). LCMS (formic) t_R = 1.36 min, *m/z* $[M + H]^+$ = 429, 89% purity; ^{1}H NMR (400 MHz, methanol-d_4) δ = 7.46 (d, *J* = 8.3 Hz, 1H), 7.27 (dd, *J* = 1.5, 8.3 Hz, 1H), 7.12 (d, *J* = 1.5 Hz, 1H), 6.24 - 6.20 (m, 1H), 4.74 (s, 2H), 3.60 (s, 3H), 2.51 - 2.41 (m, 2H), 2.30 - 2.20 (m, 2H), 2.12 - 2.02 (m, 2H), 1.92 - 1.79 (m, 4H), 1.75 - 1.56 (m, 6H), 1.48 - 1.31 (m, 2H). NH not observed.

1-(2-(5-cyclohexyl-2-oxobenzo[*d*]thiazol-3(2*H*)-yl)acetamido)cyclohexane-1-carboxylic acid (14)

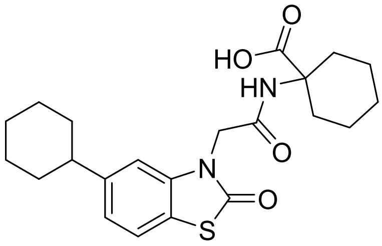

Methyl 1-(2-(5-(cyclohex-1-en-1-yl)-2-oxobenzo[*d*]thiazol-3(2*H*)-yl)acetamido)cyclohexane-1-carboxylate **36** (64 mg, 0.119 mmol) was dissolved in ethanol (40 mL) then hydrogenated using a H-cube flow hydrogenation reactor at 40 °C using a 10% palladium on carbon CatCart, 5 bar pressure and a flow rate of 1 mL/min. The solvent was evaporated in vacuo. The resulting crude product was combined with 4 M HCl in 1,4-dioxane (1.8 mL, 7.20 mmol) and water (0.5 mL), and stirred at room temperature for 3 h, then at 60 °C for 24 h. Water (0.5 mL) and 4 M HCl in 1,4-dioxane (1.8 mL, 7.20 mmol) were added, and the reaction continued to stir at 60 °C for 24 h. The reaction was then reduced in vacuo, dissolved in 1:1 MeOH/DMSO (0.8 mL) and purified by MDAP (High pH method). The desired fractions were combined and reduced in vacuo to give 1-(2-(5-cyclohexyl-2-oxobenzo[*d*]thiazol-3(2*H*)-yl)acetamido)cyclohexane-1-carboxylic acid **14** (29 mg, 0.067 mmol, 56% yield). LCMS (formic): rt = 1.27 min, *m/z* $[M + H]^+$ = 417 , 100% purity; NMR (400 MHz, DMSO-d_6) δ = 8.37 (s, 1H), 7.52 (d, *J* = 8.3 Hz, 1H), 7.07 (dd, *J* = 1.5, 8.3 Hz, 1H), 6.95 (d, *J* = 1.5 Hz, 1H), 4.62 (s, 2H), 2.56 - 2.51 (m, 1H), 2.08 - 1.93 (m, 2H), 1.85 - 1.14 (m, 18H) *COOH proton not observed.*

LCMS and NMR Traces for Key Compounds

1-(2-(6-Bromo-3-oxo-2,3-dihydro-4*H*-benzo[*b*][1,4]oxazin-4-yl)acetamido)-4,4-difluorocyclohexane-1-carboxylic acid (7)

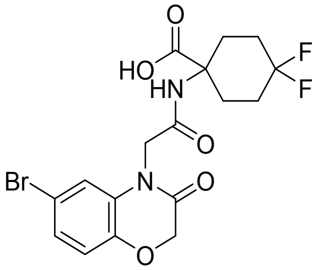

Sample: 2

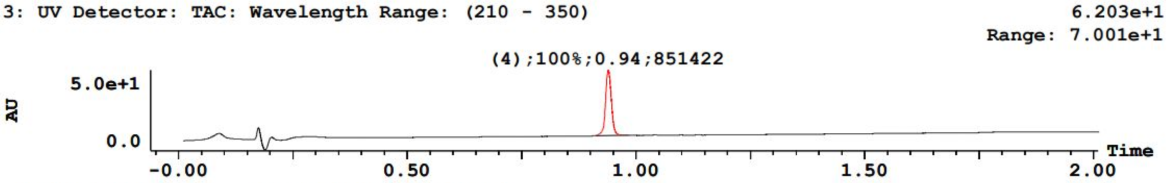

Peak Number	Time	Area %Total	Mass Found
4	0.94	100.00	Not Found

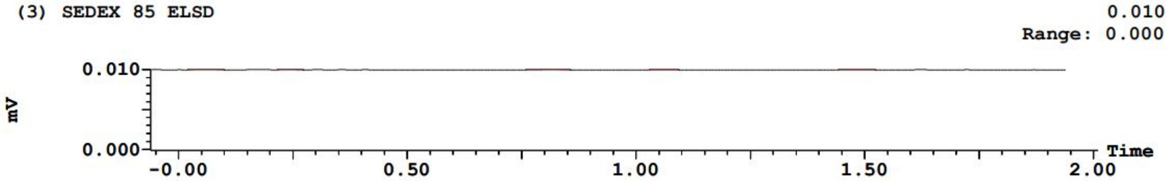

Peak	Time
4	0.94

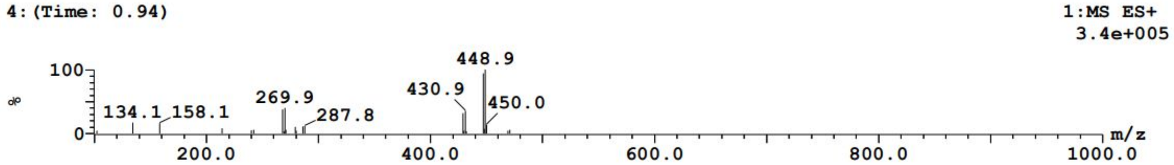

Peak	Time
4	0.94

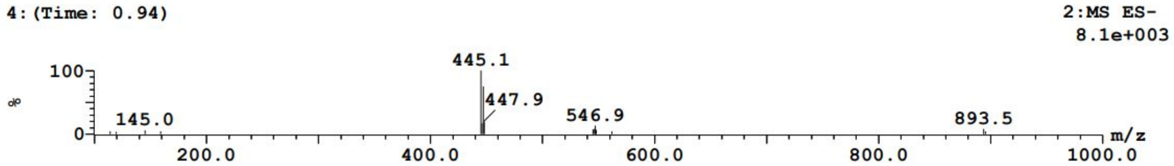

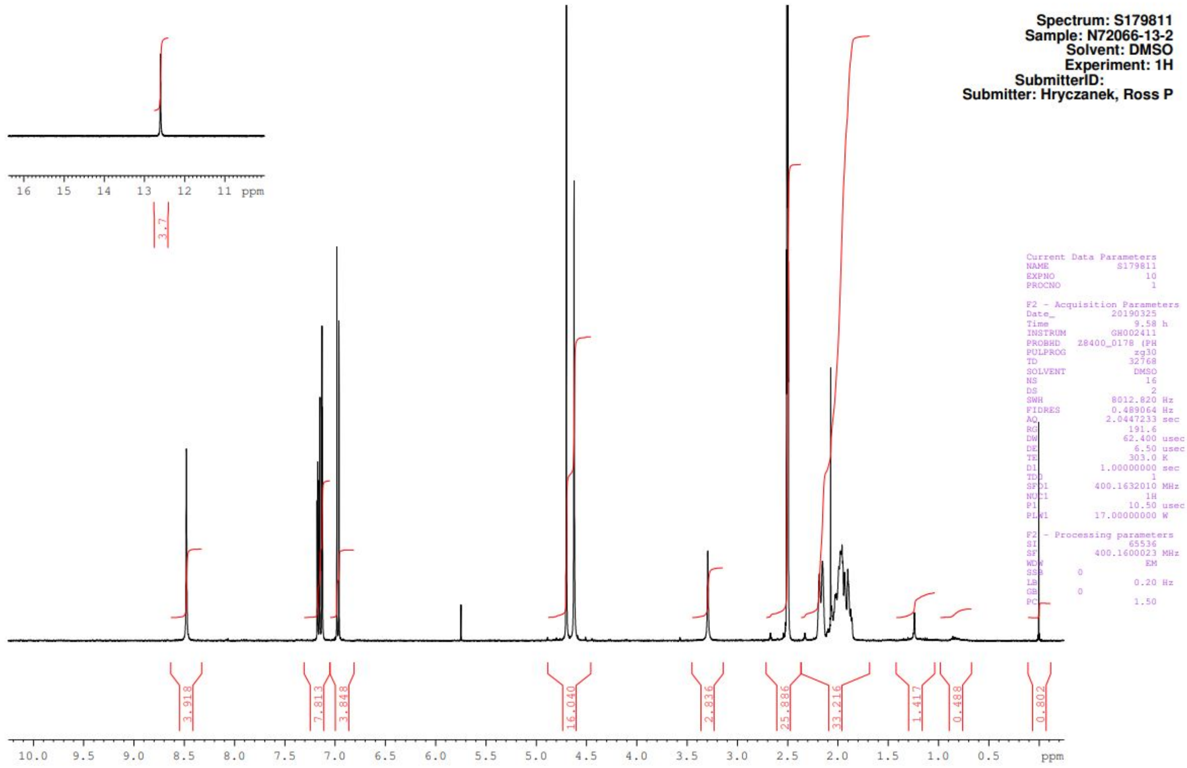
Spectrum: S179811
Sample: N72066-13-2
Solvent: DMSO
Experiment: 1H
SubmitterID:
Submitter: Hryczanek, Ross P

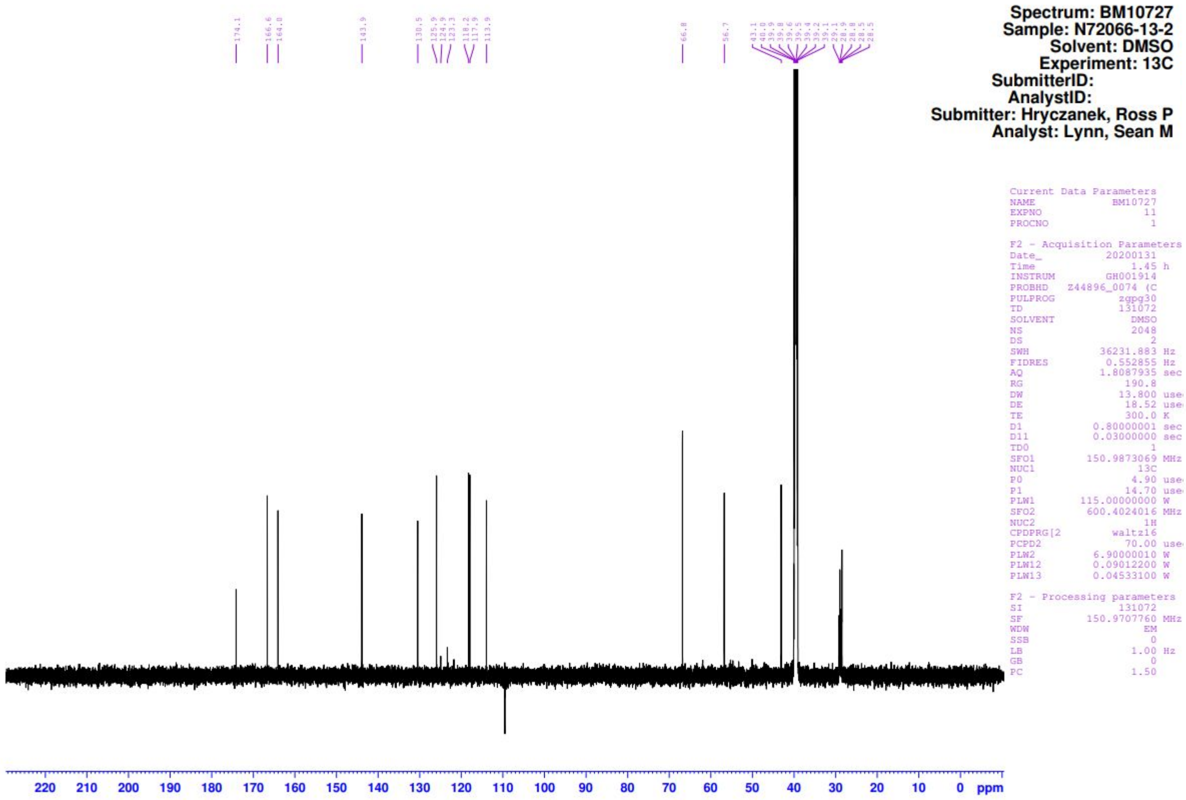
Spectrum: BM10727
Sample: N72066-13-2
Solvent: DMSO
Experiment: 13C
SubmitterID:
AnalystID:
Submitter: Hryczanek, Ross P
Analyst: Lynn, Sean M

1-(2-(2-Oxo-5-phenylbenzo[*d*]thiazol-3(2*H*)-yl)acetamido)cyclohexane-1-carboxylic acid (13)

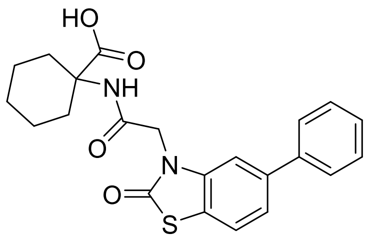

Sample: 1

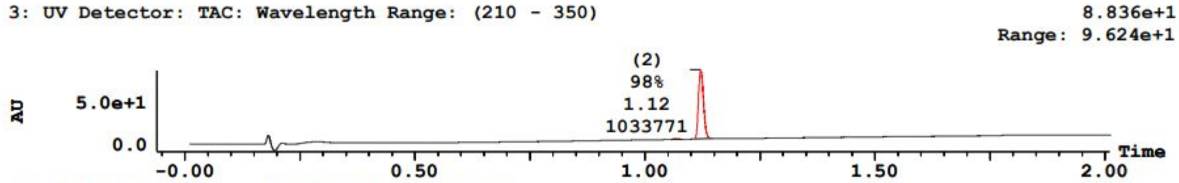

Peak Number	Time	Area %Total	Mass Found
1	1.07	1.54	Not Found
2	1.12	98.46	Not Found

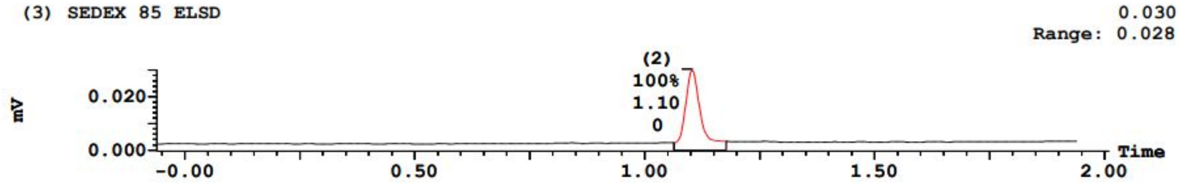

Sample Report (continued):

Peak	Time
2	1.12

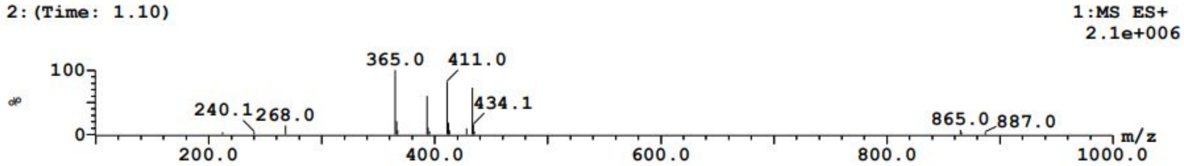

Peak	Time
2	1.12

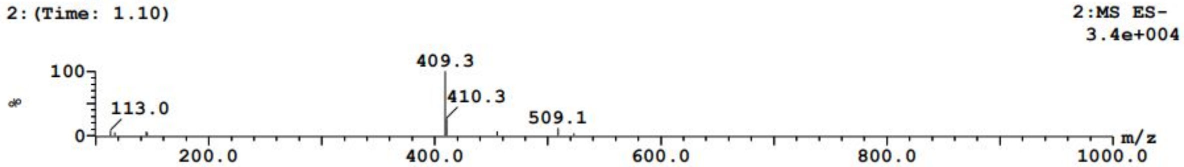

Peak	Time
2	1.12

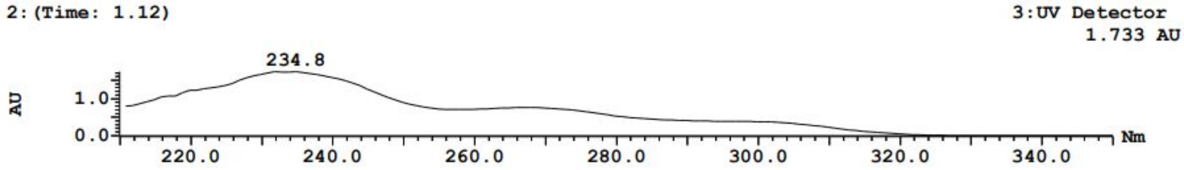

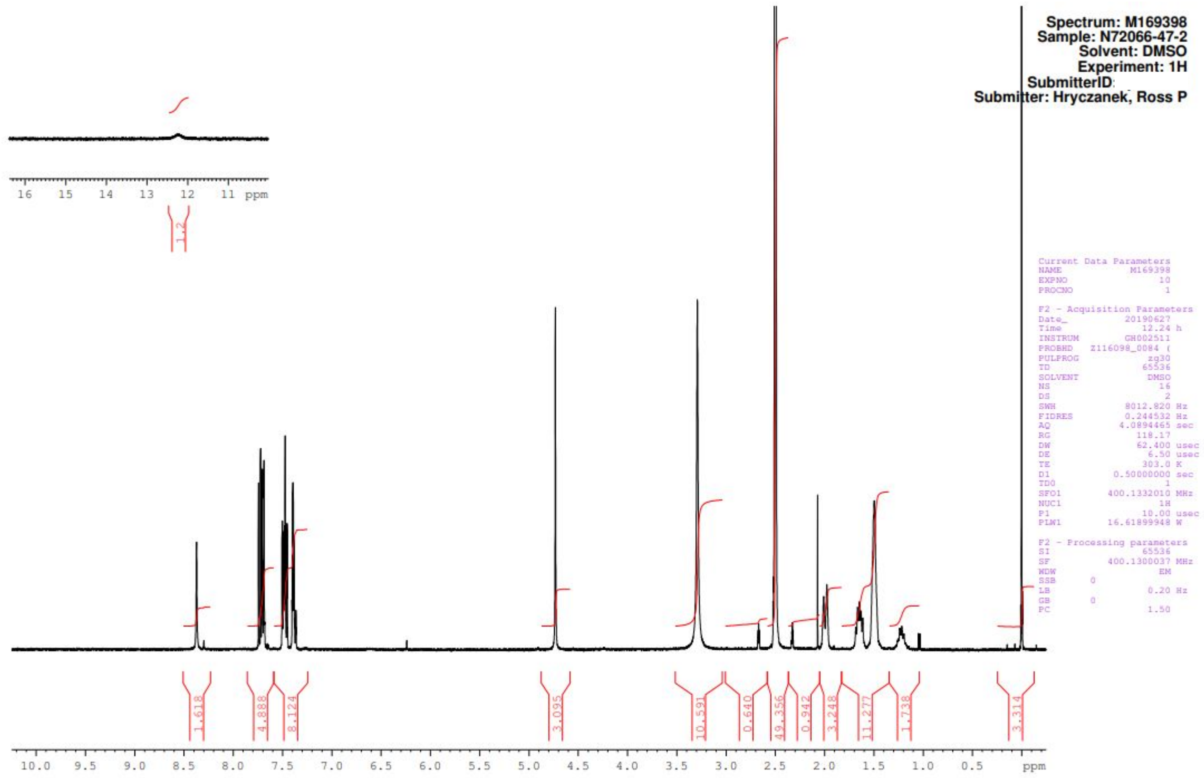
Spectrum: M169398
Sample: N72066-47-2
Solvent: DMSO
Experiment: 1H
SubmitterID:
Submitter: Hryczanek, Ross P

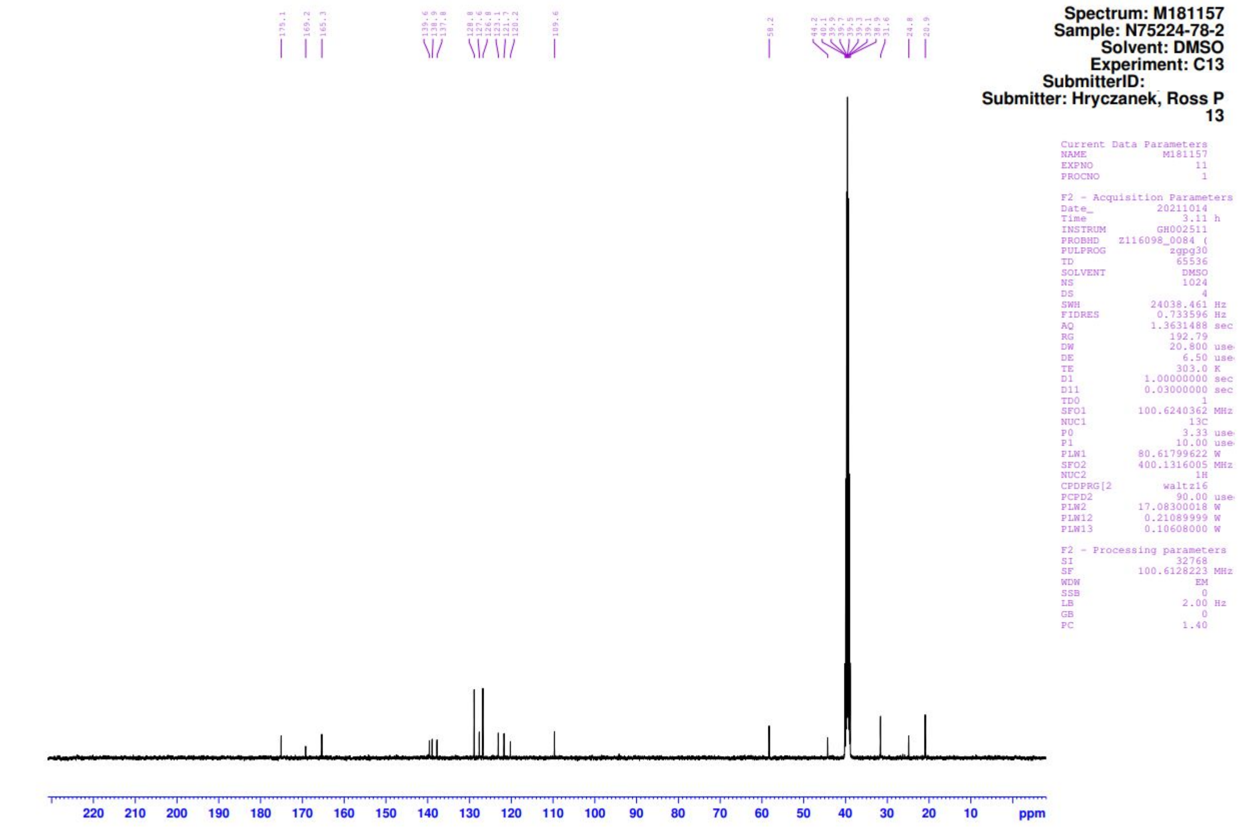
Spectrum: M181157
Sample: N75224-78-2
Solvent: DMSO
Experiment: C13
SubmitterID:
Submitter: Hryczanek, Ross P
13

Cellular Antigen Presentation Assay

The cellular antigen presentation assay was run as described previously,[1] with minor updates to the data normalisation method, see updated summary below.

The cell-based assay used to evaluate the effect of the compound on antigen presentation in cells is based on specific recognition of the ovalbumin epitope SIINFEKL when presented by the mouse MHCI H-2Kb by the antibody 25.D1.16 conjugated with allophycocyanin (APC). The assay was run in a 96-well plate format, using Cellstar 96U TC plates (Greiner), and read on an Intellicyt iQue Screener Plus flow cytometer. Two BacMam vectors, one encoding a precursor version of SIINFEKL (sequence LEQLESIINFEKL) and another encoding mouse MHC-I (H-2Kb and beta2m), were incubated with HeLa cells and compound for 40 h. Peptides encoded by the BacMams were translocated into the ER by means of signal peptide sequences. The LEQLESIINFEKL precursor was processed by endogenous ERAP1, resulting in the removal of the two N-terminal leucine residues and generation of SIINFEKL, which then bound onto H-2Kb/beta2m complexes and were translocated to the cell surface for detection by the APC-conjugated antibody. APC fluorescence was recorded at 665 nm after excitation at 533 nm. The LEQLESIINFEKL and BacMam vector also encoded GFP on a separate cassette, which was used as a marker of successful BacMam transduction and protein expression. Cell viability was evaluated using the Zombie Violet dye using excitation at 405 nm and emission at 450 nm. A threshold of 95% cell viability was used to determine DMSO tolerance, which was set to 0.5% DMSO. For normalization of the data the following equation was used: signal = 100-100(compound – control 2)/(control 1 – control 2), where control 1 is cells transduced with LEQLESIINFEKL + DMSO and control 2 is cells transduced with LEQLESIINFEKL + Control ERAP1 inhibitor (50µM FAC). Normalized data were fit to a four-parameter variable slope dose-dependence model, used to calculate PIC_{50} values.

Assay Standard Deviations and Replicates

Table S1. ERAP1 enzymatic assay

Compound	ERAP1 pIC_{50}	Standard Deviation	*n*	Notes
**1**	5.5	0.12	6	
**2**	6.7	0.23	5	
**3**	7.1	0.07	4	
**4**	7.3	0.29	4	
**5**	7.3	0.11	6	
**6**	7.9	0.11	3	
**7**	7.7	0.06	5	
**8**	7.3	0.23	3	
**9**	7.9	0.17	4	
**10**	6.6	0.13	3	
**11**	8.1	0.11	3	Tested >8.8 on one additional test occasion
**12**	8.2	0.15	4	
**13**	8.6	0.08	3	Tested >8.8 on two additional test occasions
**14**	8.2	0.1	3	

NT – not tested

Table S2. ERAP1 HeLa cell antigen presentation

Compound	HeLa Cell Antigen Presentation pIC_{50}	Standard Deviation	*n*	Notes
**1**	<4.3	null	4	
**2**	NT			
**3**	5.4	null	1	Tested <4.3 on two additional test occasions
**4**	6	0.12	2	
**5**	6	0.31	4	Tested <4.3 on one additional test occasion
**6**	6.3	0.05	2	
**7**	7	0.47	6	
**8**	6.3	0.18	4	
**9**	NT			
**10**	NT			
**11**	6.7	0.23	2	Tested <4.3 on two additional test occasions
**12**	6.7	0.01	2	
**13**	7.7	0.42	3	
**14**	7.0	0.40	3	

NT – not tested

X-Ray Crystallography Methods

The ERAP1 (1-941) Δ486-513 GSG insert protein was generated as described previously.[2] Two variants (528K or 528R) were used based on protein availability. Crystallisation was carried out using sitting-drop vapour diffusion at 20°C, with 100 + 100 nl and 100 + 50 nl (protein + well) drops. Co-crystallisation was carried out with each ligand using 100 mM DMSO stock solutions. The crystallisation conditions were initially identified from PACT screen (Qiagen) and optimised in each case, with the conditions used to grow the harvested crystals as follows. Compound **1** - 528K, 11.9 mg/ml, ~1 mM ligand, 0.2 M NaBr, 20% w/v PEG 3350, 0.1 M Bis-Tris propane pH 7.5. Compound **2** - 528R, 10.0 mg/ml, ~1 mM ligand, 25% w/v PEG 1500, 0.1 M MMT buffer pH 5.0. Compound **7** - 528R, 8.1 mg/ml, ~2 mM ligand, 22% w/v PEG 1500, 0.1 M SPG buffer pH 5.4. Compound **13** - 528R, 7.7 mg/ml, ~2 mM ligand, 24% w/v PEG 1500, 0.1 M SPG buffer pH 5.5. Crystals were harvested into a 20% ethylene glycol cryoprotectant for a few seconds and then flash frozen in liquid nitrogen.

X-ray diffraction data were collected at 100 K at the European Synchrotron Radiation Facility (ESRF) beam line ID30A-1 (Compound **1**), Advanced Photon Source (APS) beam line 22-ID (Compound **7**) and Diamond Light Source (DLS) beam line I04-1 (Compound **2**), beam line I03 (Compound **13**). The data were processed and scaled using AUTOPROC,[3] utilising XDS[4] (Kabsch, 2010), AIMLESS[5] and the CCP4 suite of programs.[6] The structures were determined using the coordinates of an isomorphous unliganded model of ERAP1 (unpublished). Preliminary refinement was carried out using AUTOBUSTER.[7] The primary ligands were clearly visible in the resulting F_o-F_c electron density maps (Figure S1). Model building was carried with COOT,[8] using a ligand dictionary generated from GRADE (Smart *et al.*, 2011). Final refinement was carried out using AUTOBUSTER. Data collection statistics and refinement details for the final models are given in Table S3. The coordinates and structure factors have been deposited in the Protein Data Bank and the accession codes will be released on publication.

ERAP1 electron density maps in the compound binding sites

Electron density F_o-F_c difference maps contoured at 3σ (green) and -3σ (red) superposed on the refined protein-inhibitor complex structures. Maps calculated after refinement of the unliganded model.

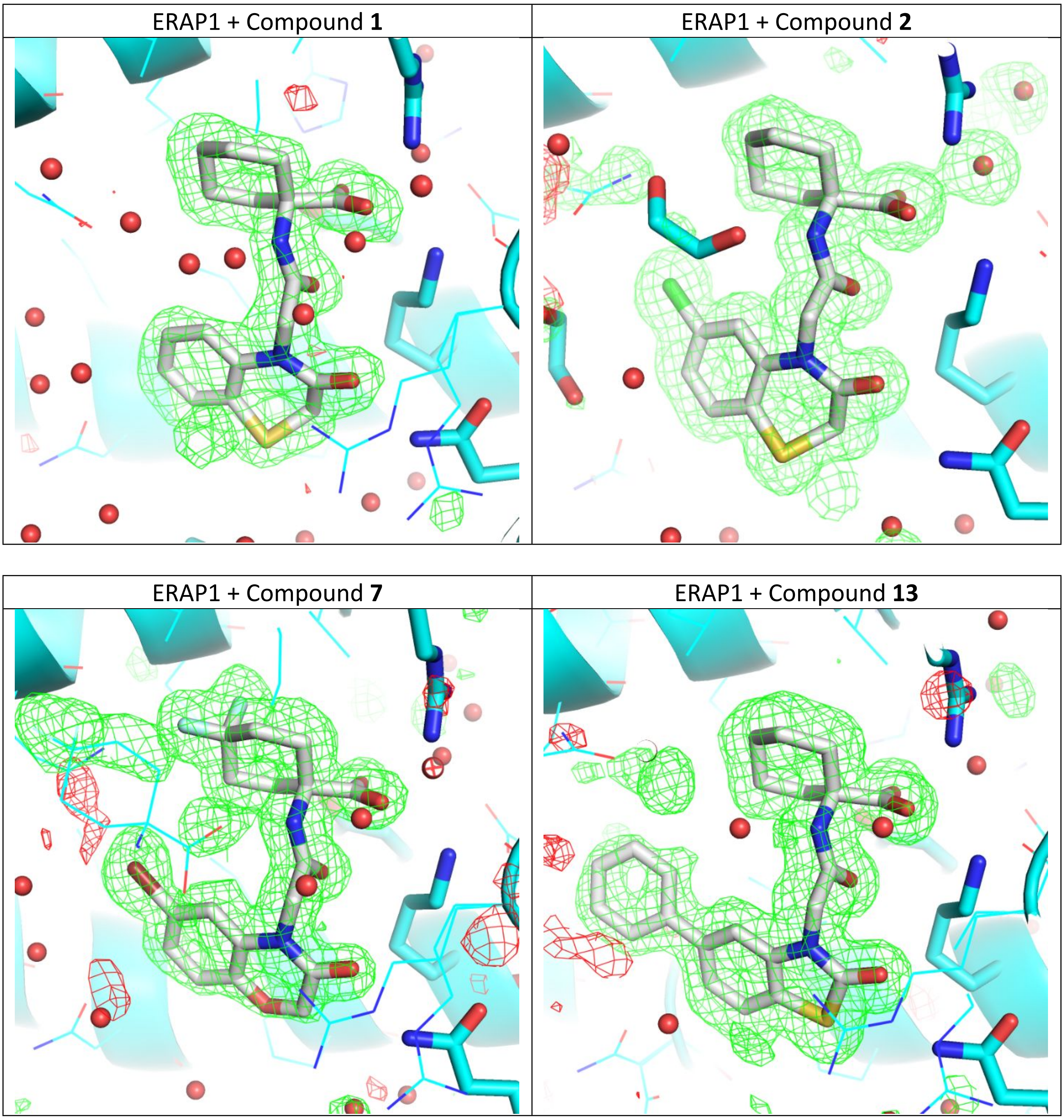

Figure S1. ERAP1 electron density maps of compound **1**, **2**, **7** and **13**

Table S3. ERAP1 data collection and refinement statistics

	Compound 1	Compound 2	Compound 7	Compound 13
**Data collection**				
Space group	$P2_1$	$P2_12_12$	$P2_12_12$	$P2_12_12$
Cell dimensions				
a, *b*, *c* (Å)	57.5, 145.4, 112.9	112.7, 140.6, 57.5	112.7, 140.8, 57.7	112.8, 140.4, 57.7
α, β, γ (°)	90, 93.3, 90	90, 90, 90	90, 90, 90	90, 90, 90
Resolution (Å)	113-1.72 (1.79-1.72)	58-1.33 (1.37-1.33)	88-1.35 (1.47-1.35)	88-1.37 (1.45-1.37)
Observations	444575 (21771)	1277445 (56697)	922371 (37855)	2283173 (119753)
Unique reflections	149277 (7466)	199598 (9980)	142076 (7104)	167841 (8395)
R_{meas}	0.097 (0.870)	0.094 (1.137)	0.120 (1.486)	0.062 (1.606)
$CC_{1/2}$	0.996 (0.559)	0.998 (0.657)	0.997 (0.327)	1.000 (0.719)
$I/\sigma I$	8.9 (1.6)	9.9 (1.4)	9.8 (1.7)	20.1 (1.6)
Completeness				
Spherical (%)	75.9 (33.8)	94.7 (56.4)	70.7 (15.9)	86.7 (26.2)
Ellipsoidal (%)	85.8 (69.3)	95.5 (60.7)	84.5 (42.7)	96.0 (58.5)
Redundancy	3.0 (2.9)	6.4 (5.7)	6.5 (5.3)	13.6 (14.3)
**Refinement**				
Resolution (Å)	113-1.72 (1.75-1.72)	58-1.33 (1.35-1.33)	20-1.35 (1.42-1.35)	88-1.37 (1.43-1.37)
R_{work}	0.169 (0.260)	0.172 (0.253)	0.171 (0.268)	0.191 (0.211)
R_{free}	0.208 (0.308)	0.190 (0.265)	0.203 (0.301)	0.207 (0.256)
No. atoms	15811		8118	7937
Protein	13914	7050	6992	7122
Ligand	141	108	89	71
Water	1756	1230	1037	744
B-factors (Å^2)				
Protein	25.1	19.3	21.2	23.2
Ligand	30.3	14.1	28.8	25.6
Water	38.8	35.7	38.6	33.6
R.m.s deviations				
Bond lengths (Å)	0.009	0.012	0.011	0.008
Bond angles (°)	0.95	1.15	1.07	0.95

*Highest resolution shell shown in parentheses

FEP Methods

Free energy perturbation simulations were performed using FEP+ (Schrodinger Small Molecule Discovery Suite v. 2018-4). The X-ray crystal structure of compound **2** in ERAP1 was used as the receptor model. The receptor model was prepared for simulation using the Protein Preparation Wizard with the following steps performed: addition of hydrogen atoms, optimization of hydrogen bonding networks and restrained minimization of all atoms. Ligand ideas were prepared for simulation using LigPrep, with protonation states and tautomers generated using Epik at pH 7.4 ± 0. Input ligand poses for FEP+ were obtained by docking to the prepared receptor model using GlideSP. A maximum common substructure core restraint was applied to maximise overlap to compound **2**. All crystallographic water molecules were retained for the FEP+ simulations with the exception of those overlapping with the docked ligand ideas. Missing ligand torsion parameters were generated using the Force Field Builder. All ligand atoms were treated as "hot atoms" for the Replica Exchange Solute Tempering sampling in the solvent leg simulations whereas the default selection was used for the complex leg. Grand Canonical Monte Carlo sampling was performed to optimize the water network surrounding the ligands. Perturbation maps were generated automatically using FEP+ with full cycle closure. The measured potency of compound **2** was used as a reference for calculating the binding affinities of other ligands. Validation of FEP+ was performed on this target and series prior to prospective use by performing a benchmarking study on series analogues of known binding affinities. All simulations were performed for 5ns.

DMPK Methods

In Vitro

Fraction Unbound in Blood

Control blood from Wistar Han Rats was obtained on the day of experimentation from in house GSK stock animals. The fraction unbound in blood was determined using rapid equilibrium dialysis technology (RED plate (Linden Bioscience, Woburn, MA) at 1 & 0.5µM for Compound **7** and **14**, respectively. Blood was dialyzed against phosphate buffered saline solution by incubating the dialysis units at 37 °C for 4 h. Following incubation aliquots of blood and buffer were matrix matched prior to analysis by LC−MS/MS. The unbound fraction was determined using the peak area ratios in buffer and in blood.

***In Vitro* Hepatocytes**

Compounds were incubated at 0.5 µM in Williams Medium E with hepatocytes of each species at 0.5 million cells/mL. The incubation plate was kept in a 37°C incubator in the presence of 5% CO2 with constant shaking at 200 rpm. Aliquots were collected at time points between 0 and 240 minutes. The resultant samples were extracted by protein precipitation with acetonitrile containing an analytical internal standard. The samples were analysed by LC-MS/MS and elimination rate constant for loss of compound from the incubation mixtures (k) was calculated from the slope of the log-transformed analyte:internal standard peak area ratio versus time curve. Data was then scaled using in house scaling factors to mL/min/g tissue.

Caco-2 Permeability

Compound 7 - Caco-2 cell monolayers were grown to confluence on PET membranes in 96-well plates. The permeability assay buffer was HBSS containing 25 mM HEPES and 25 mM glucose at a pH of 6.5 on the donor (apical) side and 7.4 on the receiver (basolateral) side. The dosing solutions contained 3 µM test compound and 5 µM elacridar (P-glycoprotein inhibitor) in the assay buffer. Cell monolayers were dosed on the apical side (A-to-B) and incubated at 37°C with 5% CO2 in a humidified incubator. Samples were collected from the donor and receiver chambers at 90 minutes. Each determination was performed in duplicate. Samples were extracted by protein precipitation with acetonitrile and were analyzed by LC-MS/MS.

Compound 13 - Caco-2 cell monolayers were grown to confluence on PET membranes in 24-well plates. The permeability assay buffer was HBSS containing 25 mM HEPES and 25 mM glucose at a pH of 7.4. The dosing solutions contained 5 μM test compound and 2 μM elacridar (P-glycoprotein inhibitor) in the assay buffer. Cell monolayers were dosed on the apical side (A-to-B) and incubated at 37°C with 5% CO2 in a humidified incubator. Samples were collected from the donor and receiver chambers at 120 minutes. Each determination was performed in triplicate. Samples were extracted by protein precipitation with acetonitrile and were analyzed by LC-MS/MS.

In Vivo

Rat Pharmacokinetic (PK) Studies

All animal studies were ethically reviewed and conducted in-house or at GSK approved preferred suppliers in accordance with the GSK Policy on the Care, Welfare, and Treatment of Animals. In brief, male Wistar Han rats were surgically prepared with a femoral vein (for drug administration) and jugular vein (for blood sampling) cannula. Rats received preoperative antibiotic and analgesics and were allowed to recover for at least 2 days prior to dosing, with free access to food and water throughout.

Intravenous (IV) PK

Rats received a 1mg/kg (1h IV infusion, 4mL/kg) solution dose of either Compound **7** (n=3) or Compound **13** (n=1) formulated in DMSO and 20% (w/v) KLEPTOSE HPB in saline aq (2%:98% (v/v)) or DMSO and 10% (w/v) KLEPTOSE HPB in saline aq (5%:95% (v/v)), respectively. Serial blood samples (c.a. 70 µL) were collected up to 24 h after the start of the IV infusion. Following collection, aliquots (20 µL) of bloodwere diluted with an equal volume of water, mixed, prior to freezing on cardice and storage at -80°C until bioanalysis.

Oral PK

Rats were orally dosed with either Compound **7** (n=3) at a target dose of 3 mg/kg (10 mL/Kg) or Compound **13** (n=1) at a target dose of 2mg/kg (10 mL/Kg), formulated as suspensions in 1% (w/v) methylcellulose aq.. Serial blood samples (c.a. 70 µL) were collected up to 24 h post oral administration. Following collection, aliquots (20 µL) of blood were diluted with an equal volume of water, mixed, prior to freezing on cardice and storage at -80°C until bioanalysis.

Sample processing and PK data analysis

Aliquots of diluted whole blood (50:50) were analysed using quantitative high-performance liquid chromatography with tandem mass spectrometric detection (LC-MS/MS) following protein precipitation. All analytical runs met predefined run acceptance criteria. Pharmacokinetic parameters were estimated from the blood concentration–time profiles using noncompartmental analysis with WinNonlin (Pharsight, Mountain View, CA).

Physicochemical Property Data

Data was acquired using published protocols: FaSSIF,[9] ChromLog$D_{7.4}$[10] and CAD aqueous solubility[11]

References

1. Liddle, J.; Hutchinson, J. P.; Kitchen, S.; Rowland, P.; Neu, M.; Cecconie, T.; Holmes, D. S.; Jones, E.; Korczynska, J.; Koumantou, D.; Lea, J. D.; Nickels, L.; Pemberton, M.; Phillipou, A.; Schneck, J. L.; Sheehan, H.; Tinworth, C. P.; Uings, I.; Wojno-Picon, J.; Young, R. J. and E. Stratikos, Targeting the Regulatory Site of ER Aminopeptidase 1 Leads to the Discovery of a Natural Product Modulator of Antigen Presentation *J. Med. Chem*., **2020**, *63* (6), 3348-3358.
2. Giastas, P.; Neu, M.; Rowland, P.; Stratikos, E., High-Resolution Crystal Structure of Endoplasmic Reticulum Aminopeptidase 1 with Bound Phosphinic Transition-State Analogue Inhibitor. *ACS Med. Chem. Lett.* **2019,** *10* (5), 708-713.
3. Vonrhein, C.; Flensburg, C.; Keller, P.; Sharff, A.; Smart, O.; Paciorek, W.; Womack, T.; Bricogne, G., Data processing and analysis with the autoPROC toolbox. *Acta crystallographica. Section D, Biological crystallography* **2011,** *67* (Pt 4), 293-302.
4. Kabsch, W., XDS. *Acta crystallographica. Section D, Biological crystallography* **2010,** *66* (Pt 2), 125-32.
5. Evans, P. R.; Murshudov, G. N., How good are my data and what is the resolution? *Acta crystallographica. Section D, Biological crystallography* **2013,** *69* (Pt 7), 1204-14.
6. Winn, M. D.; Ballard, C. C.; Cowtan, K. D.; Dodson, E. J.; Emsley, P.; Evans, P. R.; Keegan, R. M.; Krissinel, E. B.; Leslie, A. G.; McCoy, A.; McNicholas, S. J.; Murshudov, G. N.; Pannu, N. S.; Potterton, E. A.; Powell, H. R.; Read, R. J.; Vagin, A.; Wilson, K. S., Overview of the CCP4 suite and current developments. *Acta crystallographica. Section D, Biological crystallography* **2011,** *67* (Pt 4), 235-42.
7. Bricogne G.; Blanc E.; Brandl M.; Flensburg C.; Keller P.; Paciorek W.; Roversi P.; Sharff A.; Smart O. S.; Vonrhein C.; and Womack T. O. **(2017)**. BUSTER version .11.7. Global Phasing Ltd., Cambridge, U.K.
8. Emsley, P.; Lohkamp, B.; Scott, W. G.; Cowtan, K., Features and development of Coot. *Acta crystallographica. Section D, Biological crystallography* **2010,** *66* (Pt 4), 486-501.
9. Sou, T.; Bergström, C. A. S., Automated assays for thermodynamic (equilibrium) solubility determination. *Drug Discov. Today Technol.* **2018,** *27*, 11-19.
10. Camurri, G.; Zaramella, A., High-Throughput Liquid Chromatography/Mass Spectrometry Method for the Determination of the Chromatographic Hydrophobicity Index. *Anal. Chem.* **2001,** *73* (15), 3716-3722.
11. Robinson, M. W.; Hill, A. P.; Readshaw, S. A.; Hollerton, J. C.; Upton, R. J.; Lynn, S. M.; Besley, S. C.; Boughtflower, B. J., Use of Calculated Physicochemical Properties to Enhance Quantitative Response When Using Charged Aerosol Detection. *Anal. Chem.* **2017,** *89* (3), 1772-1777.
